# Motor thalamus integration of cortical, cerebellar and basal ganglia information: implications for normal and parkinsonian conditions

**DOI:** 10.3389/fncom.2013.00163

**Published:** 2013-11-11

**Authors:** Clémentine Bosch-Bouju, Brian I. Hyland, Louise C. Parr-Brownlie

**Affiliations:** ^1^Department of Anatomy, Otago School of Medical Science, University of OtagoDunedin, New Zealand; ^2^Brain Health Research Centre, Otago School of Medical Science, University of OtagoDunedin, New Zealand; ^3^Department of Physiology, Otago School of Medical Science, University of OtagoDunedin, New Zealand

**Keywords:** motor thalamus, basal ganglia, motor cortex, cerebellum, Parkinson’s disease, LTS burst

## Abstract

Motor thalamus (Mthal) is implicated in the control of movement because it is strategically located between motor areas of the cerebral cortex and motor-related subcortical structures, such as the cerebellum and basal ganglia (BG). The role of BG and cerebellum in motor control has been extensively studied but how Mthal processes inputs from these two networks is unclear. Specifically, there is considerable debate about the role of BG inputs on Mthal activity. This review summarizes anatomical and physiological knowledge of the Mthal and its afferents and reviews current theories of Mthal function by discussing the impact of cortical, BG and cerebellar inputs on Mthal activity. One view is that Mthal activity in BG and cerebellar-receiving territories is primarily “driven” by glutamatergic inputs from the cortex or cerebellum, respectively, whereas BG inputs are modulatory and do not strongly determine Mthal activity. This theory is steeped in the assumption that the Mthal processes information in the same way as sensory thalamus, through interactions of modulatory inputs with a single driver input. Another view, from BG models, is that BG exert primary control on the BG-receiving Mthal so it effectively relays information from BG to cortex. We propose a new “super-integrator” theory where each Mthal territory processes multiple driver or driver-like inputs (cortex and BG, cortex and cerebellum), which are the result of considerable integrative processing. Thus, BG and cerebellar Mthal territories assimilate motivational and proprioceptive motor information previously integrated in cortico-BG and cortico-cerebellar networks, respectively, to develop sophisticated motor signals that are transmitted in parallel pathways to cortical areas for optimal generation of motor programmes. Finally, we briefly review the pathophysiological changes that occur in the BG in parkinsonism and generate testable hypotheses about how these may affect processing of inputs in the Mthal.

## Introduction

Motor thalamus (Mthal) encompasses thalamic nuclei that are strategically located between motor areas of the cerebral cortex and two subcortical networks, the basal ganglia (BG) and the cerebellum, generally considered to be related to the complex cognitive and proprioceptive control of movement, respectively (Middleton and Strick, 2000). Lesion studies indicate that the Mthal has a role in maintaining posture, general movements and motor learning (Bornschlegl and Asanuma, 1987; Canavan et al., 1989). The main current paradigm for understanding information processing in thalamic nuclei comes from studies in sensory thalamus, and centers on the concept of contrasting functions of different inputs characterized as “drivers” and “modulators” with specific anatomical characteristics (Sherman and Guillery, 1998, 2006). However, there is considerable debate in the literature to determine what are the drivers and modulators in the Mthal, which raises the question whether this organization maps directly onto Mthal. Here, we first review the principal anatomical features and known physiology of Mthal, and the driver/modulator concept as derived from sensory thalamus. We then address whether existing anatomical and physiological evidence for cortical, BG and cerebellar inputs is consistent with driver/modulator functions, or if not, what the role of these inputs might be. We propose a new integrated model, in which cortical, cerebellar and BG afferents are considered to be of similar importance in determining Mthal activity. In this new model, Mthal acts as a “super-integrator” of motor information converging from cortex and BG, and from cortex and cerebellum, rather than simply a relay of driver signals as is thought to occur in the sensory thalamus. Finally, we consider how the different functions of inputs might impact thalamic processing in the generation of the symptoms of Parkinson’s disease (PD). By laying a platform of current knowledge about the Mthal and then speculating on a new way of thinking we aim to encourage debate and renewed experimental attention to this disregarded area of neuroscience research.

## The motor thalamus

### Anatomical organization of Mthal

Mthal is well conserved across vertebrates indicating that it is likely to play an important role in the control of movement. In mammals, it is represented by a relatively consistent region of the ventral thalamus, strongly interconnected with cerebral motor cortex, and receives extensive afferent inputs from prominent motor related structures such as the cerebellum and BG. In birds, the equivalent region according to connectivity is in the medial nucleus of the dorsolateral region (DLM; Medina et al., 1997; Luo and Perkel, 1999a,b), but the ventral location is highly conserved across many mammalian species. There are two functional subdivisions of Mthal, the BG and cerebellar receiving territories, that are also relatively conserved across species with some specific differences. In cats, four regions are generally distinguished; ventral anterior (VA), anterior and posterior subdivisions of the ventral lateral region (VLa, ventral lateral anterior and the VLp, ventral lateral posterior) and ventral medial (VM) nuclei. In rats, anatomical distinction between VA and VL is more difficult, and these are often considered together (VA/VL). However, recent studies have found molecular markers able to more easily distinguish VA and VL nuclei in rats based on their afferents (Kuramoto et al., 2011; Nakamura et al., 2012). In humans and other primates, Mthal is further subdivided into numerous nuclei and the nomenclature is not yet consistent (Hirai and Jones, 1989; Krack et al., 2002; Helmich et al., 2012). To enable data to be compared across mammalian species and nomenclatures, we have used the VA, VM, VLa and VLp scheme, and applied Mthal sub-regions to it guided by Krack et al. (2002, see their Table 1).

Mthal is interconnected with the cerebral cortex, and receives major inputs from the deep cerebellar nuclei, namely the dentate and interposed nucleus, and from the output nuclei of BG, namely the substantia nigra pars reticulata (SNpr) and internal segment of the globus pallidus (GPi). Mthal also receives major input from the reticular thalamic nucleus (Pare et al., 1987; Hazrati and Parent, 1991) and to a lesser extent, from the superior colliculus (Sommer, 2003), pedunculopontine nucleus (Steriade et al., 1988), and somatosensory spinal cord (Jones, 2007). In this review we focus on its main afferents from the cortex, cerebellum and BG.

The vast majority of Mthal neurons are glutamatergic and project out of the nucleus onto dendrites of pyramidal neurons of layers I and II and to a lesser extent layer V of the cerebral cortex (McFarland and Haber, 2002; Hooks et al., 2013). In cats and monkeys, a small population of GABAergic interneurons exists, but not in rodents (Arai et al., 1994; Jones, 2007). Mthal output neurons have a distinctive “bushy shape” with medium sized and rounded somata, and dense, circular dendritic arborizations about 300–500 μm in diameter (Williams and Faull, 1987; Kultas-Ilinsky and Ilinsky, 1991; Yamamoto et al., 1991; Sawyer et al., 1994a). Mthal neurons also have a low spine density on both distal and proximal dendrites (Sawyer et al., 1994a).

**Figure 1:**
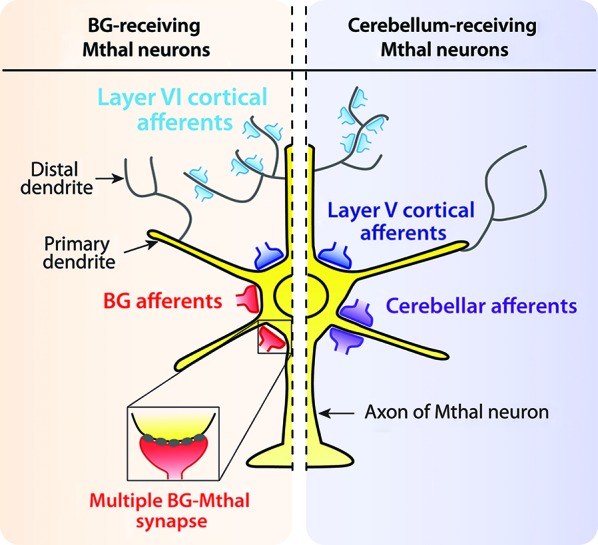
**Synaptic organisation of cortical, BG and cerebellar afferents on Mthal neurons**. Schematic diagram summarizing afferent inputs onto Mthal neurons (yellow) in BG-receiving (orange background) and cerebellum-receiving (purple background) territories. Afferents from the cerebral cortex (blue) innervate Mthal neurons in both BG (left) and cerebellar (right) receiving territories. Afferents from layer V of the cortex (large dark blue terminals) innervate somatic and perisomatic areas. Conversely, cortical layer VI afferents (small light blue terminals) innervate distal dendrites. In contrast, BG afferents (red terminals) innervate somatic and perisomatic areas of Mthal neurons, only in the BG receiving territory. The inset shows a multiple synapse formed by BG inputs. Cerebellar afferents (purple terminals) are located on primary dendrites of Mthal neurons in the cerebellar-receiving territory.

At the cellular level, cortical inputs from motor and motor-related areas innervate all Mthal neurons, whereas Mthal neurons receive inputs from either the BG or cerebellum (Ueki et al., 1977; Ueki, 1983; Yamamoto et al., 1984; Nambu et al., 1988, 1991). Mthal neurons receive cerebellar and BG afferents primarily on proximal dendrites, whereas cortical afferents terminate differently according to their laminar origin (Figure 1). Layer V cortical neurons innervate proximal dendrites, and layer VI neurons preferentially innervate distal dendrites (Kakei et al., 2001; Kultas-Ilinsky et al., 2003). The proximal location of cerebellar, BG and layer V cortical inputs indicate they are all likely to have a powerful effect on Mthal activity but the role of distal inputs from layer VI on Mthal activity is less clear.

**Figure 2:**
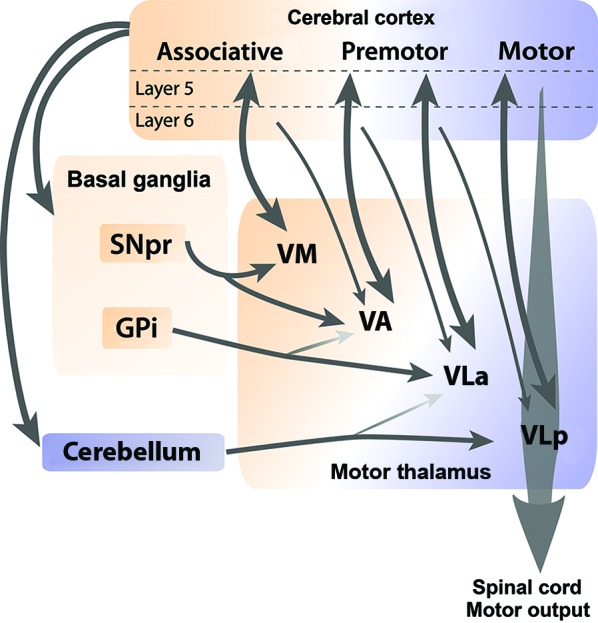
**Cortical, BG and cerebellar connections with Mthal**. Schematic diagram illustrates anatomical connections between the cerebral cortex, BG and cerebellum, and individual nuclei of the Mthal. Reciprocal connections between layer V of the cortex and Mthal nuclei are indicated by thick double-headed arrows, whereas thin arrows indicate modulator afferent inputs from layer VI of the cortex to Mthal nuclei. The ventromedial (VM) nucleus receives inputs from SNpr and associative areas of the cortex. Major inputs to the ventroanterior (VA) nucleus are from SNpr and premotor areas of the cortex, and minor inputs are from globus pallidus internus (GPi) and associative areas of the cortex. Major inputs to the anterior region of the ventrolateral nucleus (VLa) are from GPi and premotor cortex and to a lesser extent from the cerebellum. Primary inputs to the posterior region of the ventrolateral nucleus (VLp) nucleus are from the motor cortex and cerebellum, with a minor input from premotor cortex. The VLp nucleus receives inputs from collateral axons arising in layer V neurons in the primary motor cortex that descend the spinal cord. Note, (1) although some regions receive inputs from both BG and cerebellum, these two afferents do not overlap at the neuronal level and (2) that inputs from layer VI of one cortical region reach Mthal regions that are also innervated from layer V of another cortical region, allowing for integration of different cortical functions. The orange-purple color gradient represents the associative to motor continuum that exists in the cortex, BG, cerebellum and Mthal.

At the level of Mthal territories, the cerebral cortex innervates all Mthal nuclei. In contrast, BG and cerebellar afferents segregate along a rostrocaudal continuum, with GABAergic inputs from BG more rostral and cerebellar glutamatergic inputs more caudal (Figure 2). This rostrocaudal continuum is conserved across mammals, but is easier to distinguish in cats and monkeys than in rodents. Afferents from SNpr are found mainly in VA and VM nuclei, afferents from GPi preferentially target the VLa nucleus, and afferents from the cerebellum are concentrated in the VLp (Anderson and Devito, 1987; Sakai et al., 1996; Kuramoto et al., 2011; Nakamura et al., 2012). Consequently, Mthal neurons appear unlikely to directly integrate information from BG and cerebellum because these two afferents do not converge at the neuronal level or within a territory.

Connections between the cortex and Mthal form reciprocal or non-reciprocal loops depending on the laminar origin of cortical pyramidal neurons. Projections from layer V neurons of associative, premotor and motor cortical areas to Mthal are generally reciprocated (Rouiller et al., 1999; Sakai et al., 2000; McFarland and Haber, 2002; Fang et al., 2006), while layer VI axons from cortex diffusely target Mthal neurons that do not project back to the same area, but do project to other cortical regions (Rouiller et al., 1998; Kakei et al., 2001; McFarland and Haber, 2002; Haber and Calzavara, 2009). Inputs from different cortical regions are also segregated to some extent relative to the BG and cerebellar receiving areas (Anderson and Devito, 1987; Percheron et al., 1996; Rouiller et al., 1998; McFarland and Haber, 2002; Akkal et al., 2007; Haber and Calzavara, 2009; Kuramoto et al., 2009). Mthal territories receiving from BG (VM, VA and VLa) are mainly interconnected with associative and premotor cortices, whereas the cerebellar receiving territory (VLp) is preferentially interconnected with primary motor areas of the cortex (Figure 2).

It is likely that the complex connectivity between the Mthal and its afferents is important for processes related to movement preparation to be efficiently transformed to final motor commands in the motor cortex. However, the exact mechanism of the transfer of information in this network from associative to motor territories in the Mthal is not yet fully understood. One current model is that thalamic nuclei are involved in open feedback loops that facilitate integration of information coding preparatory and performance aspects of movement by “spiraling” information, first from limbic areas to non-motor thalamic nuclei (mediodorsal), thence to associative cortex, then, via Mthal to motor cortex (McFarland and Haber, 2002; Haber and Calzavara, 2009). This hypothesis is consistent with studies showing that the reaction time in rats and monkeys is about 300 ms (Baunez et al., 1995; Kurata, 2005), allowing time for development and refinement of the motor programme within corticothalamic connections. A testable prediction of this anatomically-based hypothesis is that the onset of movement-related activity during preparation for movement should be earlier in VM and VA nuclei than VLa and VLp, but no studies have addressed this point. Although it is not explicit in this theory, anatomical evidence also suggests a possible reverse transfer from motor to premotor and associative areas via the same thalamic structures (Rouiller et al., 1998, 1999; Kakei et al., 2001; McFarland and Haber, 2002; Fang et al., 2006), which may have an important feedback role for motor learning.

### Physiology of Mthal neurons

Recordings from thalamic neurons in anesthetized animals are characterized by large amplitude, slow oscillations in membrane potential with bursts of action potentials during up-states (Connelly and Errington, 2012; Ushimaru et al., 2012). This bursty activity is mainly due to the intrinsic capacity of thalamic neurons to exhibit high frequency bursts of spikes, called low threshold calcium spike (LTS) bursts, following a prolonged hyperpolarization of the membrane potential. This fundamental property of thalamic neurons relies on T-type calcium channels that have distinct dynamics (Jahnsen and Llinas, 1984a,b; Huguenard and McCormick, 1992; McCormick and Huguenard, 1992).

**Figure 3:**
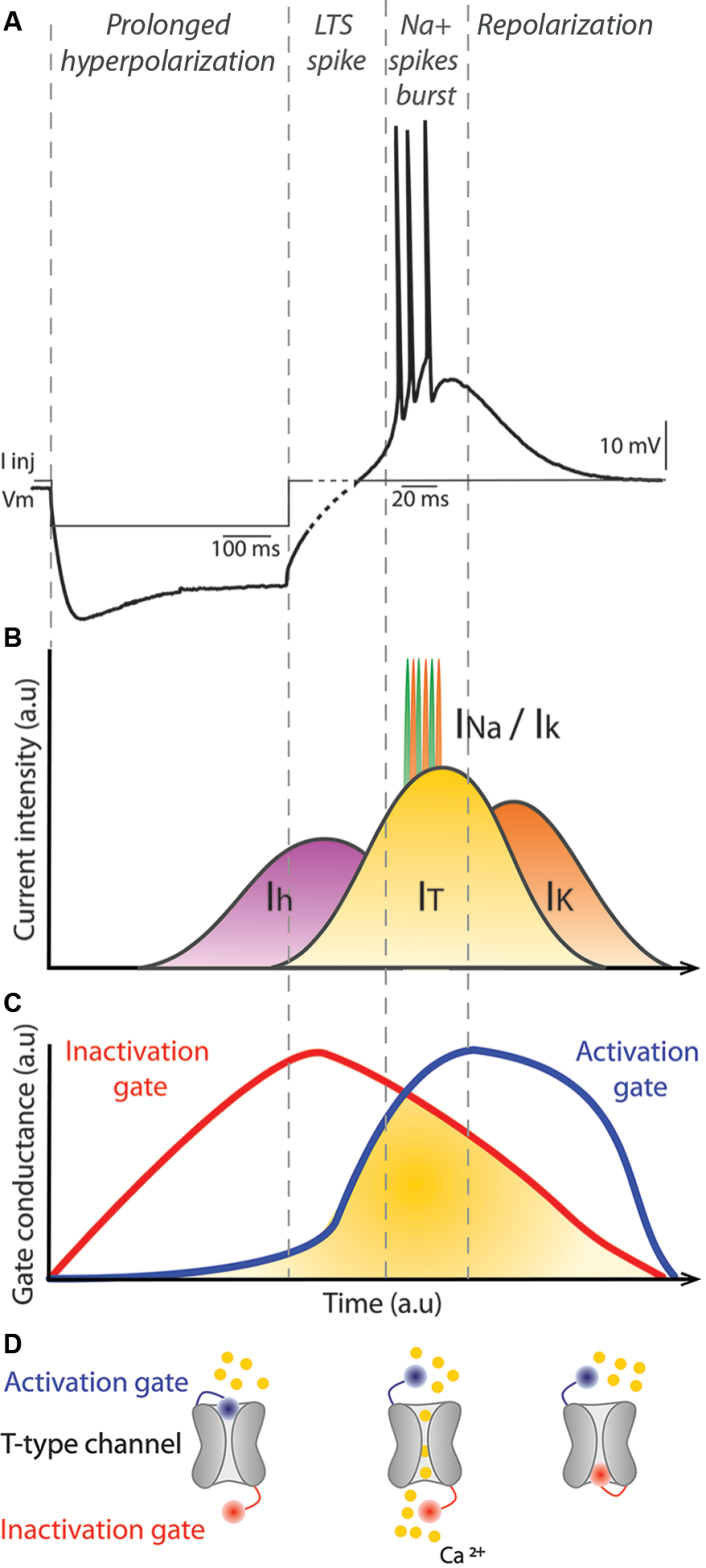
**Mechanism of LTS bursts in thalamic neurons**. **(A)** Membrane potential of a thalamic neuron recorded in current-clamp during and after injection of negative current (I Inj), which hyperpolarizes the membrane. There is a prominent sag associated with continued injection of the negative current. At the offset of the injected current, the membrane potential depolarizes and a short burst of action potentials is evoked. Note, to clearly illustrate the spikes in the LTS burst different time scales are used for the first hyperpolarization phase, prior to the dashed part of the trace (100 ms scale bar) and the spiking phase (20 ms scale bar). **(B)** Diagram represents the major conductances underlying the membrane potential changes shown in **(A)**. The I_h_ cationic current is activated by hyperpolarization of the membrane potential, which depolarizes the membrane potential and is the current mainly responsible for the sag. When the neuron repolarizes after current injection, the I_T_ current is activated, which augments depolarization of the membrane potential. When the threshold for voltage-gated sodium channels is reached, action potentials occur and they are represented in **(B)** by the successive I_NA_ and I_K_ currents. Progressively, the I_T_ current is reduced, which favors activation of I_K_ currents and the neuron is repolarized to its resting membrane potential. **(C)** Conductances associated with inactivation (red) and activation (blue) gates of the T-type calcium channel underlying the I_T_ current. Once the membrane potential is hyperpolarized, the inactivation gate opens slowly. As the membrane potential depolarizes, the inactivation gate closes slowly and at the same time the activation gate is opened. When both gates are open, the I_T_ current occurs. Finally, the activation gate closes as the membrane potential returns to rest. **(D)** Illustration of a T-type calcium channel with its activation (blue) and inactivation (red) gates. Calcium ions are represented in yellow. The left example illustrates the configuration of the channel when the neuron is hyperpolarized. The middle and right examples illustrate the channel configuration when the I_T_ current occurs and when the neuron is at its resting membrane potential, respectively. a.u: arbitrary units.

The T-type calcium channels in the thalamus are depolarizing channels that are activated following a prolonged hyperpolarization of the membrane potential under −70 mV (Figure 3). This initial prolonged hyperpolarization is necessary to de-inactivate the channel (Figure 3), thus making the channel responsive to depolarizing events. When the membrane is depolarized following a prolonged period of hyperpolarization, the T-type channel is activated (opens) briefly and the influx of calcium ions (I_T_ calcium current) further depolarizes the membrane leading to activation of voltage-gated sodium channels underlying the generation of action potentials (Figure 3). Because the membrane potential of Mthal neurons remains depolarized by the T-type calcium channels for a relatively long duration (∼50 ms), the prolonged depolarized state triggers multiple spikes in the LTS bursts. The T-type channel then inactivates and the gate closes, permitting repolarization of the membrane potential (Figure 3). LTS burst activity is a characteristic firing pattern in the thalamus in certain brain states and has raised much interest in the field of thalamus physiology since its discovery. LTS bursts are frequently observed during slow wave activity (Hirsch et al., 1983; Llinas and Steriade, 2006) or when the animal becomes drowsy (Joffroy and Lamarre, 1974; Strick, 1976; Schmied et al., 1979). As far as we are aware, the occurrence of LTS bursts in the Mthal within a complete BG-thalamocortical network in awake mammals has only been reported in an abstract (Postupna and Anderson, 2002), therefore, the role of LTS bursts in awake states remains to be fully characterized.

A rapid sequence of spikes, such as occurs in LTS bursts, has different consequences on network activity than a single spike and these modes may encode specific aspects of information, depending on the system and/or the context (Xu et al., 2012). In the brain, the probability of neurotransmitter release at synapses by a single spike is generally low (Borst, 2010; Tarr et al., 2013). With a burst of spikes, the probability of neurotransmitter release is augmented considerably, increasing the reliability of synaptic transmission (Lisman, 1997). This is true for both LTS bursts, and other kinds of bursts elsewhere in the brain that are not triggered by a prolonged inhibition. One function of LTS bursts during awake states may be to trigger state changes such as between inattentive rest and active movement (Crick, 1984; Sherman, 2001; Bezdudnaya et al., 2006). Thus, while tonic firing seems to dominate Mthal activity during awake states, LTS bursts may occur at a particular moment in time to increase reliability of synaptic transmission to downstream neurons in the cortex. LTS bursts may also contribute to the plasticity of responses in thalamic neurons (Hsu et al., 2012) as they are triggered by a strong influx of calcium, a fundamental activator of synaptic and intrinsic plasticity (Xia and Storm, 2005).

Despite extensive studies about the role and mechanisms of LTS bursts in the sensory thalamus, very little data exist on LTS bursts in Mthal. Interestingly, in the songbird homolog of Mthal (DLM), LTS bursts can be triggered by GABAergic synaptic events coming from Area X (equivalent to GPi in mammals), in the *in vitro* slice preparation (Luo and Perkel, 1999a; Person and Perkel, 2005). Although it remains unknown if this is true in mammalian brains, it raises the possibility that GABAergic BG inputs may be able to have an excitatory effect on Mthal neurons, as discussed in more detail in section entitled Characteristics of Cortical, Cerebellar and BG Afferents in the Mthal.

### Activity of the Mthal and its main afferents during motor behavior

Mthal is defined as “motor” because of the extensive inputs from motor cortex, BG, and cerebellum; brain regions that exhibit changes in activity related to, and are essential for, preparation and execution of movements. Motor cortex and its associated cortical areas exhibit changes in spiking activity related to the parameters of the movement, such as velocity, orientation or force (Thach, 1978; Georgopoulos et al., 1982, 1983; Kalaska et al., 1983; Georgopoulos et al., 1986; Georgopoulos, 1988; Kalaska et al., 1989; Caminiti et al., 1990; Wickens et al., 1994; Quallo et al., 2012). The dominant current theory is that all parameters of the movement are coded in the motor cortex by disparate subpopulations of pyramidal neurons that may be functionally synchronized during preparation and execution of a movement by brief oscillatory patterns to form the motor programme (Sanes and Donoghue, 1993; Vaadia et al., 1995; Baker et al., 1997; Churchland et al., 2012). Consequently, neurons recorded individually in the motor cortex exhibit complex and variable movement-related modulations in activity.

Deep cerebellar nuclei, and the dentate nucleus in particular, display mainly increases in activity, preceding the movement or associated with the visual cue (Thach, 1978; Mink and Thach, 1991; Mushiake and Strick, 1993; Horne and Butler, 1995; Middleton and Strick, 2000; Ebner et al., 2011). In contrast to the motor cortex, there remains considerable debate on whether the cerebellum specifically codes parameters of a movement such as direction or amplitude (Trouche and Beaubaton, 1980; Mink and Thach, 1991; Thach et al., 1992; Horne and Butler, 1995; Ebner et al., 2011). Neural changes in movement-related activity in cerebellar nuclei are generally thought to coordinate movements across multiple joints, have a role as a temporal pattern generator, code proprioceptive information and error signalling to optimize movements and motor learning possibly through feed-forward and/or adaptive filter models, but do not specifically control initiation of movements (Sieb, 1989; Thach et al., 1992; Horne and Butler, 1995; Braitenberg et al., 1997; Ohyama et al., 2003; Jacobson et al., 2008; Dean et al., 2010; D’Angelo et al., 2011; Ebner et al., 2011).

The BG output nuclei, GPi and SNpr, display a variety of changes in activity related to movement, with GPi seemingly more related to movement execution whereas SNpr neurons tend to change their activity in relation to the preceding cue and the movement-related reward (Nambu et al., 1990; Mink and Thach, 1991; Jaeger et al., 1995; Mushiake and Strick, 1995; Turner and Anderson, 1997, 2005; Wichmann and Kliem, 2004; Nambu, 2007; Nevet et al., 2007; Fan et al., 2012). Like the cortex, BG output nuclei code direction and amplitude of movements (Georgopoulos et al., 1983; Turner and Anderson, 1997; Turner and Desmurget, 2010). At a more complex level, activity in the BG is dramatically modified between the first and last trials of a motor learning task (Jog et al., 1999; Barnes et al., 2005; Fan et al., 2012; Lemaire et al., 2012), indicating that it may play a role in motor learning. The BG receiving territory of Mthal is thus ideally situated to be involved in motor learning because of its connections with prefrontal cortex and premotor cortex (McFarland and Haber, 2002; Xiao et al., 2009; Redgrave et al., 2010).

Neuronal recordings in the Mthal of awake animals display a wide range of activity from low to high frequencies (1–80 Hz), with brief modulations in activity in relation to movements (Anderson and Turner, 1991; Forlano et al., 1993; Macia et al., 2002; Pessiglione et al., 2005). This mode of firing differs dramatically from the activity in the Mthal during slow wave activity in EEGs or local field potential recordings during anesthesia, where activity is organized in bursts that are repeated with consistent periodicity (Steriade et al., 1971; Nakamura et al., 2012). Given that the inputs to Mthal all display some movement-related modulation in activity, information processing in Mthal during preparation or execution of movement is expected to also be reflected in temporally specific modulations. The activity of Mthal neurons during motor behavior has been mainly studied in primates and in behavioral paradigms requiring a movement triggered by a cue. These studies report that Mthal neurons change their activity in the period between presentation of the cue and onset of the movement, then activity returns to baseline levels (Strick, 1976; Schmied et al., 1979; Horne and Porter, 1980; MacPherson et al., 1980; Anderson and Turner, 1991; Nambu et al., 1991; Butler et al., 1992, 1996; Forlano et al., 1993; Vitek et al., 1994; Inase et al., 1996; Ivanusic et al., 2005; Kurata, 2005). These changes are mainly increases in activity, but decreases or complex patterns are also reported. The activity of Mthal neurons is correlated with movement duration, velocity or force, but only in a minority of cells (Butler et al., 1996; Ivanusic et al., 2005). Interestingly, despite anatomical segregation of information in BG and cerebellar territories, neurons across all regions of Mthal exhibit similar ranges of firing rate and movement-related activity (Anderson and Turner, 1991; Nambu et al., 1991). This may reflect the fact that both BG output nuclei and deep cerebellar nuclei display complex responses with similar temporal characteristics during comparable tasks (Mushiake and Strick, 1993; Fan et al., 2012), but the precise role of each afferent on Mthal activity is still unknown. It can also be explained by inputs common to all motor thalamic nuclei such as afferents from premotor and motor cortices or the reticular thalamic nucleus (Pare et al., 1987; Hazrati and Parent, 1991). Another hypothesis is that glutamatergic synapses from cerebellum onto Mthal neurons are depressed and thus are unlikely to increase the firing frequency of Mthal neurons (Nakamura et al., 2012).

Insight about the role of the Mthal in the control of movement can be obtained from animal studies that have examined the effect of lesion or intrathalamic drug injection on behavior. Lesion effects are dependent on the site of the thalamic lesion, but in general these studies suggest that Mthal has a role in maintaining posture, controlling general movements and in motor learning. Mammals exhibit akinesia and bradykinesia, and posture is impaired following VA, VLa, VLp and VM electrolytic lesions or injection of GABA agonists or glutamate antagonists intrathalamically (Di Chiara et al., 1979; Starr and Summerhayes, 1983a,b; Klockgether et al., 1986a,b; Wullner et al., 1987; Canavan et al., 1989; Jeljeli et al., 2003). Large lesions of the VA, VLa and VLp nuclei produce ataxia and dysmetria in the contralateral arm of primates (Bornschlegl and Asanuma, 1987). The VLa appears to be particularly important in learning motor tasks because large lesions that included the VLa severely impaired relearning in primates, whereas lesions confined to VA did not (Canavan et al., 1989). Similarly in songbirds, the DLM is critical for motor learning, but not the production of song. DLM lesions exclusively impaired motor practice (babbling) and development of complex, mature syllables that are typical of adult male birds (Johnson and Bottjer, 1993; Goldberg and Fee, 2011).

Despite these advances, many questions remain to be addressed about the nature of information processing in Mthal and its role in motor control. In particular, Mthal activity needs to be explored during more complex motor tasks and during motor learning, and little data are available about how this activity is regulated by the various inputs. Finally, the role of Mthal in regulating activity in its efferent targets remains unknown.

## Characteristics of cortical, cerebellar and BG afferents in the Mthal

Much is known of the physiology of sensory thalamus and principles derived from sensory thalamus have been applied to Mthal because of their physical proximity and similarly intense interconnectivity with cortex. A major organizing principle for sensory thalamic nuclei is the classification of afferents as being either *drivers* or *modulators* (Sherman and Guillery, 1998, 2006). Driver afferents define the sensory receptive field properties of thalamic neurons and dictate spiking activity, whereas modulator afferents influence the activity of thalamus cells without directly triggering spikes. Activity in the sensory thalamus is thus strongly correlated with the activity of driver inputs but not with modulator inputs (Sherman and Guillery, 1998). A typical example is the lateral geniculate nucleus, which relays visual information from the retina to the visual cortex (Sherman and Guillery, 1998, 2006). Here, retinal ganglion inputs are drivers because they define the receptive field properties of relay neurons in the lateral geniculate nucleus, whereas inputs from the parabrachial region, reticular thalamic nucleus, layer VI of the cortex and local interneurons are considered modulators, because they do not fill the driver criteria (Sherman, 2007).

To determine if an input is a driver or a modulator, Sherman and Guillery (Sherman and Guillery, 1998, 2006, 2011; Sherman, 2007) defined several criteria. Basically, these criteria state that driver inputs have anatomical and physiological features that ensure information is reliably transmitted to, and controls the activity of, downstream thalamic neurons. An afferent input that does not fulfil all of these criteria is considered by default to be a modulator.

Anatomically, driver inputs have large diameter axons, with a dense terminal arborization, and preferentially target proximal dendrites and perisomatic areas of postsynaptic thalamic neurons. An additional anatomical criterion is that drivers do not send any collateral axon to the reticular thalamic nucleus. The reticular thalamic nucleus is intimately connected to the cortex and its GABAergic projection neurons innervate most thalamic nuclei, including Mthal (Pare et al., 1987; Hazrati and Parent, 1991). The significance of this additional criterion is not explicitly explained in the literature, but if a driver also sends collateral input to the reticular thalamic nucleus, feed forward inhibition from the reticular thalamic nucleus may simultaneously reduce the strength of the primary driver signal to Mthal neurons.

Although these anatomical features identify good candidate driver inputs of thalamic activity, physiological features must also be considered. For example, while it is generally assumed that afferents proximal to the cell body have a stronger effect on neuronal activity than distal ones due to degradation of the electrical signal along dendrites, this is not always the case because synaptic events can be electrically maintained by active conductances along dendrites (Gulledge et al., 2005). Four main physiological criteria characterize driver inputs (Sherman and Guillery, 1998, 2006, 2011; Sherman, 2007). First, in the sensory thalamus, it is considered that a driver has to be glutamatergic because only excitatory neurotransmitters are able to directly trigger action potentials in adult postsynaptic neurons. Second, the transmitter must act via ionotropic receptors for temporally precise and rapid onset/offset of conductances at the postsynaptic membrane. Conversely, transduction of the synaptic signal via metabotropic receptors can last for several hundreds of milliseconds, which is not compatible with temporally precise transmission of information across a synapse but could contribute to synaptic plasticity (Luscher and Huber, 2010). Third, the synaptic input has to be significant to produce large synaptic events that reliably trigger an action potential in the postsynaptic neuron. Fourth, a driver input should display paired pulse depression, which is a decrease in the amplitude of current for successive synaptic events when they are triggered at frequencies between 10 and 250 Hz. The reason for this criterion is that paired pulse depression means there is a high probability of neurotransmitter release at the first synaptic event, leading to a lower probability that a second synaptic event will be reliable (Zucker and Regehr, 2002). However, this last criterion is complex to interpret since paired pulse depression can be due to multiple factors with both pre and postsynaptic origins (Klug et al., 2012).

Several studies examining Mthal physiology have attempted to address whether cortical, cerebellar and BG afferents are more likely to be driver or modulator inputs, but the results to date are inconclusive (Anderson and Turner, 1991; Smith and Sherman, 2002; Person and Perkel, 2005; Bodor et al., 2008; Goldberg and Fee, 2012; Gulcebi et al., 2012; Nakamura et al., 2012; Rovo et al., 2012). Therefore, the following sections summarize the available anatomical and physiological data for cortical, cerebellar and BG inputs to Mthal and compare their features to the characteristics of drivers and modulators in an attempt to understand what their role might be in the Mthal. We also introduce the term “driver-like”, when afferents fulfil most, but not all, of the criteria of a driver input. Notably, whereas traditional models of thalamic function based on sensory nuclei assume that each nucleus receives only one driver input (Sherman and Guillery, 2006), this review highlights that Mthal may integrate inputs from multiple sources, including driver-like afferents from BG.

### Cortical afferents have driver and modulator characteristics, depending on the layer of origin

Cortical afferents to Mthal arise from pyramidal neurons in layers V and VI. Layer V afferents to the Mthal are collaterals from major descending axons that project to the brainstem and spinal cord. These layer V afferents, which represent a small proportion of all cortical inputs to Mthal, have been reported to match the anatomical criteria of a driver (Grofova and Rinvik, 1974; Rouiller et al., 1998; Kakei et al., 2001; Kultas-Ilinsky et al., 2003; Rouiller et al., 2003). However, a recent study did not report any large glutamatergic afferents from the cortex in the Mthal based on immunohistochemical staining for a glutamate transporter (vGLUT1), a stain for cortical driver afferents (Rovo et al., 2012). Nevertheless, layer V axons are thick (up to 3 μm) and terminate with very large boutons on the perisomatic area and proximal dendrites of thalamic neurons (Kultas-Ilinsky et al., 2003). Moreover, layer V afferents do not terminate in the reticular thalamic nucleus (Kakei et al., 2001; Kultas-Ilinsky et al., 2003). Functionally, one study reports that these layer V corticothalamic neurons from the motor cortex have a fast conduction velocity (∼40 m/s) typical of large diameter myelinated axons (Sirota et al., 2005), but further physiological data are not available and the postsynaptic receptors have not been characterized. While anatomical and physiological features of layer V afferents to Mthal are consistent with a driver role, full characterization is not yet available.

In contrast, cortical afferents from layer VI meet several modulator criteria. They preferentially target distal dendrites of Mthal neurons and have small diameter axons with small bouton terminals in the Mthal (Kakei et al., 2001; Kultas-Ilinsky et al., 2003). Moreover, layer VI cortical afferents send axon collaterals to the reticular thalamic nucleus (Kakei et al., 2001). Physiological data are scarce, but the conduction velocity of axons from layer VI cortical pyramidal neurons is less than 5 m/s, which is significantly slower than axons from layer V (Sirota et al., 2005). Nothing is known functionally, including whether synapses formed by layer VI inputs have ionotropic or metabotropic receptors. In summary, anatomical data suggest that layer VI cortical afferents to Mthal may have a less prominent role than layer V afferents.

The consequences of two functional inputs from the cortex on information processing in the Mthal during the preparation and execution of movements needs further consideration in the context of reciprocal and non-reciprocal thalamocortical connections. From the criteria described above, it appears that the features of layer V and VI afferents of Mthal activity are consistent with driver and modulator roles, respectively. There is a reciprocal feedback loop between cortical layer V and Mthal. Layer V afferents directly innervate the Mthal, and Mthal sends axons back to the corresponding cortical region (Figure 2). In contrast, layer VI afferents have an important role in integrating information across functional cortical boundaries because their axons target Mthal neurons that project to other cortical regions (McFarland and Haber, 2002; Haber and Calzavara, 2009). We can suppose from these data that Mthal activity of a specific nucleus is driven by its corresponding cortical area with layer V afferents and modulated at the same time by neighboring cortical areas with layer VI afferents, enabling the spiraling of information between the cortex and Mthal to facilitate the best functional movement outcome. In that case, layer VI cortical afferents would play a critical role in the corticothalamic network because they would be responsible for ensuring the Mthal integrates information across functional cortical areas. Currently, movement-related responses of layer VI pyramidal neurons in motor areas vary markedly (Sawaguchi et al., 1989; Matsumura et al., 1992; Beloozerova et al., 2003a,b; Isomura et al., 2009), therefore, further studies need to determine the precise role of layer VI cortical inputs on Mthal activity during execution of movements.

### Cerebellar afferents in the Mthal exhibit several driver characteristics

The connection between deep cerebellar nuclei and Mthal has been less studied than cortical afferents but the anatomical and physiological features indicate that cerebellar afferents to Mthal have several driver characteristics (Sherman and Guillery, 2006; Rovo et al., 2012). Indeed, application of the standard model developed from arrangements in sensory thalamus (where first order nuclei involve an ascending pathway whereas higher order nuclei are mainly implicated in corticocortical communications (Sherman and Guillery, 2006)), the cerebellar receiving territory of Mthal is considered to be a first order nucleus, driven by the cerebellum and modulated by layer VI cortical afferents (Sherman and Guillery, 2006; Rovo et al., 2012).

Consistent with a driver role, cerebellar afferents in Mthal form large boutons that mainly synapse on primary dendrites (Rinvik and Grofova, 1974; Kultas-Ilinsky and Ilinsky, 1991; Aumann et al., 1994; Sawyer et al., 1994b; Kuramoto et al., 2011; Rovo et al., 2012). These anatomical features are corroborated by intracellular studies showing that stimulation of cerebellar afferents produces strong, fast, excitatory events in Mthal neurons, that are even faster than cortical ones (Uno et al., 1970; Shinoda et al., 1985; Sawyer et al., 1994a). However, two important criteria of driver inputs that remain unknown are if the cerebellum innervates the reticular thalamic nucleus and the type of glutamatergic receptors involved at cerebellothalamic synapses. Further, the cerebellar receiving territory of Mthal also receives inputs from layer V of the cortex (Rouiller et al., 1998; McFarland and Haber, 2002), which, as noted above, have characteristics consistent with a driver role.

### BG afferents in the Mthal have several driver-like characteristics

BG afferents in the Mthal are from the GPi and SNpr. Although these two BG afferents do not terminate on exactly the same nuclei within Mthal, we will consider them together because they are both GABAergic and the temporal aspects of their neural activity are similar at rest and during execution of movements (Wichmann et al., 1999; Boraud et al., 2002; Wichmann and Kliem, 2004).

Currently, the role of BG inputs on Mthal activity remains an enigma. One model classifying thalamic nuclei by their inputs has proposed that the BG receiving territory of Mthal is a higher order nucleus, driven by layer V cortical afferents and modulated by BG and layer VI cortical afferents (Sherman and Guillery, 2006; Gulcebi et al., 2012). However, several anatomical and physiological features of BG inputs to Mthal are consistent with a driver-like role and an alternative model proposes that BG inputs have a strong impact on Mthal activity (Albin et al., 1989; Alexander and Crutcher, 1990).

The main argument for BG providing modulatory input is that BG projection neurons are GABAergic and thus by definition, cannot be drivers (Sherman and Guillery, 2006). Intracellular recordings of Mthal activity have demonstrated that electrical stimulation in the GPi/SNpr induces strong inhibitory synaptic events in the Mthal (Deniau et al., 1978; Uno et al., 1978; Anderson and Yoshida, 1980; Chevalier and Deniau, 1982; Ueki, 1983; Tanibuchi et al., 2009). Moreover, the membrane potential of neurons in Mthal is about −60 mV during the anesthetized state (Paz et al., 2007), some 10 mV from the reversal potential for chloride, which favors generation of an inhibitory current. In the awake state, this will be exacerbated because neurons are less hyperpolarized (Franks, 2008) with firing rates between 1 and 80 Hz (Anderson and Turner, 1991; Forlano et al., 1993; Macia et al., 2002; Pessiglione et al., 2005), making GABAergic inputs from BG more likely to hyperpolarize Mthal neurons than excite them. While it is possible for GABAergic afferents to depolarize neurons depending on the equilibrium potential for chloride, (Viitanen et al., 2010; Kim et al., 2011), to date, an excitatory effect of BG input onto Mthal neurons mediated by direct GABAergic activation of ionotropic receptors has not been found in mammals. Another argument for a modulator role of BG afferents is that the SNpr sends axons to the reticular thalamic nucleus (Pare et al., 1990; Pazo et al., 2013), although it remains unclear whether these are collaterals of axons innervating the Mthal or a different set of neurons.

In distinct contrast to the assumption of a modulator role for BG inputs that develops from a thalamic perspective, the second theory assumes that the BG exert driver-like control on Mthal activity. This theory is based on models of BG function and circuitry, which treat the BG-territory of the Mthal as a “relay” responsible for transmitting BG output to cortex (Albin et al., 1989; Alexander and Crutcher, 1990; DeLong, 1990; Boraud et al., 2002; Bar-Gad et al., 2003; Nambu, 2004). Indeed, some anatomical and physiological characteristics of BG synapses onto Mthal neurons are consistent with a driver-like role. First, the synapse between the GPi and the Mthal in cat exhibits paired pulse depression (Uno et al., 1978). Second, receptors involved in the transmission between BG and the Mthal in birds are exclusively ionotropic (Luo and Perkel, 1999a). Third, and particularly importantly, electron microscopy studies show that synapses formed by GPi and SNpr terminals onto Mthal neurons in mammals are notably large and have giant (or multiple) synapses (Grofova and Rinvik, 1974; Kultas-Ilinsky and Ilinsky, 1990; Sakai et al., 1998; Bodor et al., 2008; Kuramoto et al., 2011; Rovo et al., 2012). The term “multiple synapses” has been chosen because every SNpr individual terminal forms between 5 and 20 synaptic contacts on Mthal neurons that are closely spaced on one bouton (Bodor et al., 2008; Figure 1). These multiple synapses are also concentrated on proximal dendrites and somata of Mthal neurons (Sakai et al., 1998; Bodor et al., 2008; Rovo et al., 2012). Moreover, these synaptic contacts are not separated by astrocytes (Bodor et al., 2008), which favors GABA spillover to perisynaptic ionotropic receptors and induces much larger inhibitory currents with a tonic inhibitory conductance (Farrant and Nusser, 2005). While ionotropic receptors are not generally thought to underlie LTS burst spiking because the GABA_A_ current is too fast to cause the prolonged hyperpolarization required to deinactivate the T-type calcium channel, the multiple synapse arrangement combined with the lack of astrocytes in close proximity to the synapse, favors a large amplitude hyperpolarization that is long enough to promote generation of an LTS burst (Jahnsen and Llinas, 1984a,b). Whether an LTS burst is generated at these giant synapses in mammals is not known, but it is one of several factors, such as synchronized GABAergic BG inputs and expression of GABA_A_ versus GABA_B_ receptors on Mthal neurons, that will affect generation of LTS bursts.

The specialized synapse structure of GABAergic inputs to Mthal is even more obvious in birds. Indeed, Area X (equivalent of GPi in mammals) connects DLM neurons (equivalent of Mthal neurons in mammals) with calyx-like synapses at a 1:1 ratio, in which GABAergic multiple synaptic contacts are closely spaced and distributed all around the soma (Luo and Perkel, 1999a,b; Doupe et al., 2005). The avian brain provides a strategic advantage to examine communication between BG and Mthal because activity in the calyx-like synaptic terminals from Area X can be simultaneously recorded with the soma of DLM neurons (Luo and Perkel, 1999a,b; Doupe et al., 2005). Indeed, recording extracellular spikes in axon terminals is rare as these electrical signals are usually very small (up to a thousand times smaller) compared to somatic spikes (Hubel and Wiesel, 1961; Schomburg et al., 2012). Recordings in the avian brain show reliable transmission from BG to Mthal with some temporal specificity (Person and Perkel, 2007; Kojima and Doupe, 2009; Leblois et al., 2009; Goldberg and Fee, 2012), consistent with the idea that the calyx-like terminals from the BG can provide a driver-like input that controls Mthal spiking. However, the state of the animal may critically determine the effect of BG input on Mthal inputs. In anesthetized birds where BG inputs have high firing rates (∼100 Hz) and the firing rate of Mthal neurons is low (∼5 Hz), BG inputs dominate spiking activity in Mthal (Person and Perkel, 2007; Kojima and Doupe, 2009; Leblois et al., 2009). In contrast, in awake singing birds, when BG firing rates are very high (∼300 Hz) and firing rates in the Mthal are also high (∼100 Hz), inputs from the cortex determine spiking activity in Mthal (Goldberg and Fee, 2012). These data indicate that excitatory and inhibitory inputs may have different consequences on Mthal activity depending on pre- and postsynaptic firing rates (Smith and Sherman, 2002; Guo et al., 2008; Goldberg et al., 2012). Notably, the firing rate of thalamic neurons in the awake bird is too high (∼100 Hz) to allow deinactivation of the T-type calcium channel for LTS bursts to occur (Goldberg and Fee, 2012).

The avian BG-thalamic synapse is a special case, and it remains necessary to determine the physiological characteristics of BG inputs to Mthal in mammals to further understand how Mthal neurons process inputs, particularly in the awake state and during movement execution. Nevertheless, the available data in Mthal show that BG afferents have several characteristics that are consistent with both modulator and driver-like roles. Therefore, like cerebellum, the BG-territory of Mthal appears to receive more than one source of input that could play a driver-like role. Because of the importance of the BG-Mthal circuit in BG pathology, such as PD, we now consider in more detail the roles BG inputs may play in modulating Mthal activity.

### Control of Mthal activity by BG inputs: possible mechanisms and implications

There are currently three main mechanisms proposed for how BG could control Mthal activity; the rebound model focusing on LTS bursts, the gating model focusing on the disinhibitory role of BG inputs and the entrainment model focusing on the temporal role of BG inputs. The strongest evidence for the rebound model comes from the anesthetized or *in vitro* avian brain, where inhibition of Mthal neurons by BG inputs reliably evokes LTS bursts that are locked in time (Person and Perkel, 2005, 2007; Kojima and Doupe, 2009; Leblois et al., 2009). Through the specialized calyx-like terminal structure, these BG inputs cause prolonged hyperpolarization of Mthal neurons that trigger a rebound LTS bursts of spikes. Therefore, BG afferents can be seen as indirect excitatory inputs due to their ability to trigger LTS bursts. We hypothesize that the role of LTS bursts may be complex and context dependent, in a similar way to synchronization of neuronal populations (Baker et al., 2001), occurring at precise, discrete periods during a movement. In this sense, BG GABAergic inputs to Mthal are not only consistent with most of the anatomical and physiological criteria of a driver input, but they may have the additional function of augmenting a functional movement-related signal when they trigger a high-frequency burst of spikes. BG inputs could thus increase the reliability of the synaptic transmission of Mthal neurons to downstream neurons in the cortex because they trigger bursts of spikes temporally locked to the offset of inhibitory inputs.

The rebound model has not yet been investigated in mammals but a model study has shown that LTS bursts may allow detection of an inhibitory drive (Smith and Sherman, 2002). The few available data report that LTS bursts in the Mthal of awake primates do occur, but at very low rates (Postupna and Anderson, 2002) or particularly when animals are drowsy (Joffroy and Lamarre, 1974; Strick, 1976; Schmied et al., 1979). This may reflect the fact that there are fewer LTS bursts in freely moving animals but it may also be due to limitations in detecting LTS single spikes using extracellular recording techniques. Following a prolonged hyperpolarized state, T-type calcium channels in Mthal neurons can partially activate and trigger one spike but not necessarily a burst of spikes (Llinas and Steriade, 2006). This issue will only be resolved when the changes in membrane potential underlying all spikes are recorded in awake animals, which requires extremely challenging patch clamp or intracellular recordings in behaving animals. Given the lack of data investigating the significance of LTS bursts in Mthal activity during execution of movements, studies need to analyze neuronal recordings for LTS bursts and LTS spikes in behaving animals to understand the impact of this firing pattern on downstream structures.

Another mechanism proposed for how BG could exert powerful control on Mthal activity is the gating model (Horak and Anderson, 1984; Deniau and Chevalier, 1985; Chevalier and Deniau, 1990; Hikosaka, 2007). In this model, BG outputs can indirectly excite Mthal neurons, through disinhibition. BG are assumed to inhibit the thalamus under basal conditions because SNpr and GPi display high spontaneous spiking rates (between 10 and 70 Hz in mammals) (Wichmann and Kliem, 2004; Avila et al., 2010), releasing GABA in the Mthal. However, when the BG network is activated by cortical input, SNpr and GPi outputs are transiently suppressed and downstream targets, including the Mthal, are disinhibited.

Consideration of the complete cortico-BG network leads to a more precise formulation of the expected functional impact of BG input in this model. There are three main pathways for information transmission through the BG, the hyperdirect, direct and indirect pathways (For review, see Nambu, 2004). These pathways vary in the number of synapses from input to output, leading to the possibility that a single input signal could lead to successive waves of varying output. Thus, the hyperdirect pathway directly excites subthalamic nucleus, which in turn excites BG output nuclei, the GPi and SNpr. The direct pathway synapses in the striatum, which then inhibits the BG output nuclei, with a longer latency than the hyperdirect pathway. Finally, the indirect pathway, which synapses in both striatum and external part of the globus pallidus, leads to disinhibition of BG output nuclei, at the longest latency (Fujimoto and Kita, 1992; Maurice et al., 1998, 1999; Kolomiets et al., 2003). At the level of the GPi and SNpr, the consequence of the sequential activation of these three pathways is thus an excitation—inhibition—excitation sequence (Maurice et al., 1998, 1999; Kolomiets et al., 2003). According to the gating model, this BG output would be expected to cause a mirror sequence of Mthal activity. Following phasic input from BG, baseline firing rate in Mthal activity would first be inhibited, then disinhibited, and finally inhibited again before activity returns to baseline levels (Schneider and Rothblat, 1996; Nambu, 2004). The putative disinhibition of the Mthal produced by the direct BG pathway is the key element of the gating model. The BG may act as a gate that “decides” when cortical afferents freely drive Mthal activity.

The third mechanism to explain how BG control Mthal activity is the entrainment model. In this model, Mthal spiking is not positively correlated with BG activity, which would be expected for a driver input, but is instead restrained to a precise temporal window in which thalamic neurons can fire (Goldberg et al., 2012). It is postulated that BG input has an entrainment role because excitatory inputs that drive spiking of Mthal neurons interact with brief pauses of BG inhibition (Goldberg and Fee, 2012; Goldberg et al., 2012). This model has been extrapolated from studies in birds (Goldberg and Fee, 2012; Goldberg et al., 2012), where the activity of BG and Mthal is very high compared to mammals (∼300 and 100 Hz during singing, respectively). A comparable role of BG inputs on the temporal precision of Mthal activity has not yet been shown in mammals. However, in other brain areas receiving both glutamatergic and GABAergic inputs, interplay between the timing of these inputs has been shown to increase temporal precision of spiking activity in the post-synaptic neuron (Mainen and Sejnowski, 1995; Baufreton et al., 2005). The role of GABAergic inputs on Mthal activity needs to be explored further to determine if this temporal refining role of BG inputs applies in the Mthal of mammals.

In summary, it remains unknown if BG inputs control Mthal activity by the rebound, gating or entrainment models in mammals. The few available data indicate that the mechanism of information processing from BG to Mthal is dependent on the behavioral state of the animal, which is also supported by a study suggesting that the excitability of Mthal neurons is a critical factor for determining how the Mthal processes inputs (Goldberg et al., 2012). Therefore, to significantly advance our understanding of how the Mthal processes information underlying the control of movement, it is important that future studies are conducted in mammals executing movement tasks.

## A new theory: Mthal integrates, rather than relays, information from cortical, cerebellar and BG afferents

**Figure 4:**
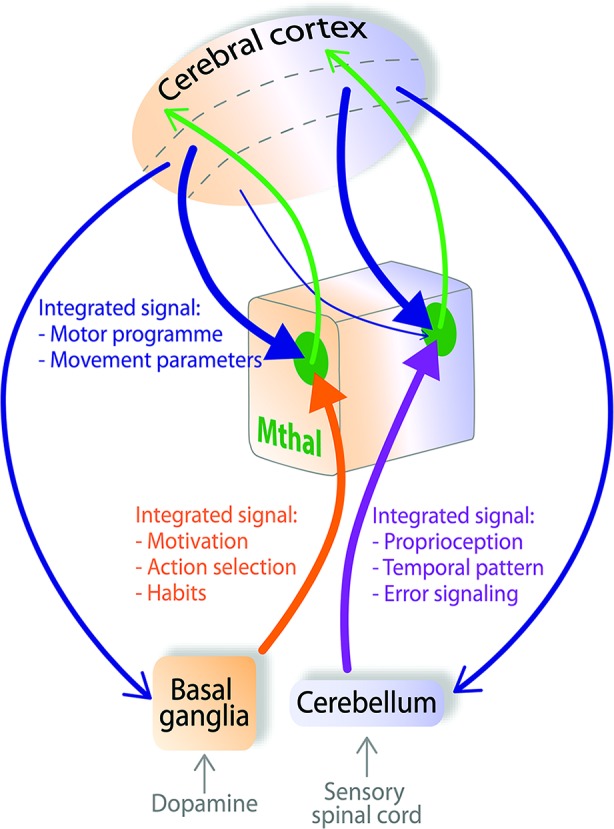
**Mthal acts as a “super integrator” of cognitive and proprioceptive information from the cortex, BG and cerebellum**. In the model proposed here, pyramidal neurons in layer V of cortical motor areas send a copy of the developing motor programme (blue arrows) simultaneously to the BG, Mthal and cerebellum. The BG integrates motivational context through inputs from the dopaminergic system. The cerebellum integrates proprioceptive context by its innervation from the sensory spinal cord. The BG (orange pathway) and cerebellum (purple pathway) contribute these separately integrated inputs to BG (orange background) and cerebellar territories (purple background) of Mthal. Arrowheads from the cortex, BG and cerebellum to the Mthal are of equal size signifying that these inputs all strongly influence Mthal activity. Modulator inputs from layer VI of the cortex (thin blue arrow) are proposed to transfer information from cortex receiving particularly strong input from one Mthal area, back to the other Mthal area. The respective Mthal areas process all converging inputs (green zones) and forward a “super-integrated” signal back to the cortex (green arrows) for development of the motor programme. Dashed lines in the cerebral cortex separate superficial layers (top), from layers V (middle) and VI (bottom).

Several features of Mthal connectivity indicate it may process information very differently to sensory thalamus. The anatomical and physiological features of inputs to Mthal are not consistent with the dichotomous driver/modulator characteristics derived from sensory thalamus. In particular, evidence suggests the presence of multiple drivers, from cortex and cerebellum in the cerebellar territory and from cortex and BG in the BG territory, as reviewed above. Therefore, we propose a new theory of information processing in the Mthal summarized diagrammatically in Figure 4, where layer V of the cortex, cerebellum and BG can all have a strong influence on Mthal activity.

In this model, Mthal acts as a “super-integrator”, actively assimilating information from multiple inputs. We propose the term “super-integrator” for Mthal because BG and cerebellar networks also receive information about the initial motor programme from the cortex, and independently integrate the cortical signal before forwarding it to their respective Mthal territories. Functionally, BG input is assumed to be responsible for adding motivational context, due to the dense dopaminergic inputs they receive, to select the best action needed to achieve the required behavioral outcome (Hassler, 1978; Redgrave et al., 2010). Similarly, cerebellar input will provide complex proprioceptive information, processed from sensory afferents of the spinal cord, vestibular apparatus etc., so that the current position of the body in space is used to optimize the motor programme (Eccles, 1973; Braitenberg et al., 1997). Figure 4 also shows that the BG and cerebellar territories of the Mthal receive a copy of the developing motor programme directly from respective functional areas of the associative, premotor and motor cortices via projections from pyramidal neurons in layer V. Mthal processes information from all of these highly integrated inputs, with the weighting of each input dependent on the context and required motor outcome. Then, projections from Mthal return highly refined “super-integrated” motor plans back to the cortex to update development of preparatory and performance parameters of the motor programme.

The model outlined above proposes that information from BG and cerebellar territories project in parallel pathways to their recipient cortical areas (see Figures 2, 4). However, we need to consider in this model that BG and cerebellar territories of Mthal exhibit broadly similar movement-related modulations in activity during preparation and execution of movements (Anderson and Turner, 1991; Nambu et al., 1991). Given the lack of overlap of cerebellar and BG input to single Mthal neurons, this uniform activity may reflect very complex integration and information processing in the Mthal such as “spiraling” of information from the cortex to Mthal, other inputs that innervate both territories, and disparate inputs having similar functional consequences on Mthal activity. As discussed in section entitled Cortical Afferents have Driver and Modulator Characteristics, Depending on the Layer of Origin and summarized in Figure 2, corticothalamic efferents originating in layer VI transfer information from one functional area of cortex in a non-reciprocal relationship to another functional area in the Mthal, for example from cerebellum receiving Mthal to BG receiving Mthal, and *vice versa*, as depicted in Figure 4. Such iterative spiraling has been proposed to be important for fine-tuning an appropriate activation pattern of cortical neuronal networks that control agonist and antagonist muscles for a movement, and to contribute to response reaction times (Wickens et al., 1994). Inputs to both BG and cerebellar territories from other afferents, notably from the reticular thalamic nucleus, but also from the pedunculopontine nucleus, superior colliculus and locus coeruleus, may contribute to the similar movement-related activity in these territories as well (Lindvall et al., 1974; Rivner and Sutin, 1981; Pare et al., 1987; Steriade et al., 1988; Hazrati and Parent, 1991; Sommer, 2003). Furthermore, as discussed in section entitled Activity of the Mthal and its Main Afferents during Motor Behavior, while BG and cerebellum differ in the precise coding of a motor plan, they also have some similar movement-related modulations in activity, probably because they both receive input from motor cortex. Thus, integration of diverse and complex inputs by the Mthal could explain similarities in movement-related activity across both territories at the gross level, but still allow fine spatiotemporal differences, which are discussed below.

Importantly, various physiological mechanisms underpin this processing. Rather than uniformly additive integration in the Mthal, specific features of the inputs mean that they could converge to either boost a signal (e.g., cortical and cerebellar glutamatergic inputs) or act competitively (e.g., cortical glutamatergic inputs and GABAergic BG afferents). Furthermore, the input-output relationship resulting from this integration will vary greatly depending on the respective timing and the firing rate of both presynaptic afferents and the postsynaptic neuron, and on brain state, accounting for the variety of features seen under different experimental conditions. For instance, such interactions could account for the fact that Mthal activity in the awake, resting state is asynchronous throughout corticothalamic, cortico-BG-thalamic and cerebellothalamic networks, while it is highly synchronized under anesthesia (Steriade, 2006; Crunelli and Hughes, 2010; Rowland et al., 2010). In awake, resting states, cerebellar and BG output neurons have the ability to generate action potentials autonomously (Eccles, 1973; Raman and Bean, 1997; Atherton and Bevan, 2005; Bosch et al., 2011), therefore, they have high spontaneous firing rates, but cortical inputs do not (Chen et al., 1996; Barth and Poulet, 2012). Input from BG and cerebellar afferents thus bombard their respective Mthal territories to prevent the cortex and Mthal from oscillating in synchrony.

In addition to the anatomical-functional considerations that underpin integration in the Mthal, as summarized in Figure 4, it is also important to consider temporal aspects of inputs and link these to spatial aspects to fully understand how the patterns of Mthal activity might arise. These temporal-spatial dimensions are likely to be important for determining the precise contribution BG and cerebellar Mthal territories make to motor programme development. With respect specifically to BG inputs, as reviewed in section entitled Control of Mthal Activity by BG Inputs: Possible Mechanisms and Implications, there is considerable evidence in support of a gating model in which temporal organization of activity in specific afferent pathways plays a crucial role; in particular, it has been proposed that Mthal is first inhibited by the hyperdirect pathway, then disinhibited by the direct BG pathway (Maurice et al., 1998, 1999; Kolomiets et al., 2003). This is followed by a further period of inhibition, mediated by the indirect BG pathway. Such serial events could underpin temporally precise coding of activity of specific individual muscles. In addition, there is also a superimposed spatial dimension to Mthal processing. Neurons in BG output nuclei define multiple parallel channels of motor information (Hoover and Strick, 1993), and in support of this idea, there is a lack of correlated activity between SNpr neurons during a cue-reward movement task (Nevet et al., 2007). These parallel channels of motor information from BG will also be reflected in the spatial location of Mthal neurons whose activity is modulated in relation to a movement, and each channel could have its own specific temporally organized pattern of activity on these separate BG pathways. Thus, we postulate that each Mthal neuron or small group of neurons receives a unique, integrated signal from BG output nuclei, and temporal and spatial mechanisms enhance the contrast between motor information to be promoted and suppressed in the Mthal. This could allow coding of temporally precise interrelationships of activity across different muscle groups, represented by spatially defined Mthal micro-domains.

The integrating role proposed for the Mthal is in stark contrast to the driver/modulator dichotomy and the relay function of sensory thalamic nuclei. This is perhaps not surprising given the different requirements that are likely to apply to motor and sensory functions, which would be expected to be reflected in the way these nuclei process information. Sensory thalamic nuclei need to relay information that accurately reflects stimulation of sensory receptors so that an animal correctly perceives their environment. In this case, relaying information through the sensory thalamus to the cortex with little integration will ensure that the neural code most accurately reflects stimuli at the original sensory receptor. This is achieved by each sensory thalamic nucleus having one defined driver that determines the overall activity in this nucleus, whereas all other afferents are modulators that fine-tune the neural activity. In contrast, for optimal motor function, a complex set of contextual information needs to be integrated so that the best motor programme can be activated. BG and cerebellar territories of the Mthal provide key sites to enable that to occur because they receive motivational and complex proprioceptive information about the movement that have been integrated in the BG and cerebellum and will enhance activation of the motor programme to be promoted and suppress unwanted motor programmes. For the Mthal to effectively select the best motor programme, it cannot simply relay the information from one driver input. Instead, the precise spatial and temporal pattern of activation in Mthal will reflect a functional movement goal.

## Information processing in the Mthal in Parkinson’s disease

PD is characterized by three major motor symptoms; rigidity, slowing of movements and tremor at rest. In this section, we focus on the possible roles of BG inputs in the Mthal because BG activity is profoundly altered in PD and although the cerebellum is a major input to Mthal, there is a paucity of data reporting changes in PD.

PD is caused by the progressive degeneration of dopaminergic neurons from the substantia nigra pars compacta that innervate the striatum and the other BG nuclei (Albin et al., 1989). Because dopamine plays a crucial role in BG physiology, the dynamics of the BG network are profoundly altered in the PD condition (Obeso et al., 2000; Boraud et al., 2002; Walters et al., 2007). Given that the BG are a major afferent of Mthal, understanding Mthal pathological activity is likely to be central to fully understand the neurophysiological origins of PD symptoms.

From the numerous studies that have examined BG activity in PD model animals and patients, the key pathological features of neural activity across BG nuclei can be summarized as follows:

1. an irregular and bursty pattern of activity in BG nuclei, whereas spiking activity is more regular and tonic in basal conditions in control animals (DeLong, 1971; Hassani et al., 1996; Tai et al., 2003; Parr-Brownlie et al., 2007). This is particularly clear in the external part of the globus pallidus, the subthalamic nucleus and the two output nuclei, the GPi and SNpr.
2. an increase in synchronization between neurons within a BG nucleus and between BG nuclei, compared to a relatively unsynchronized activity in control animals (Nini et al., 1995; Raz et al., 2000, 2001; Brown et al., 2001; Dejean et al., 2008; Brazhnik et al., 2012).
3. an increase in beta oscillations (12–30 Hz) found in all BG nuclei compared to control animals (Hutchison et al., 2004; Sharott et al., 2005; Avila et al., 2010). Brief periods of beta oscillations are present in normal conditions, but power in the beta range is exaggerated in amplitude and duration in the parkinsonian state (Jenkinson and Brown, 2011).
4. a loss of specificity in the “receptive fields” of BG neurons (Filion et al., 1988; Bergman et al., 1994; Abosch et al., 2002; Guehl et al., 2003). Individual neurons are more likely to respond to passive movements around multiple joints and in multiple directions than in control conditions.

Cortex has been relatively less studied in PD animal models, but changes in bursty activity and synchronization (Goldberg et al., 2002; Parr-Brownlie and Hyland, 2005; Pasquereau and Turner, 2011) and loss of specificity (Goldberg et al., 2002) have also been found in the primary motor cortex, which is both an afferent to and recipient of inputs from the Mthal. Moreover, it appears that motor cortex activity is more synchronized with BG nuclei such as the subthalamic nucleus, striatum or SNpr, in PD than in control conditions (Magill et al., 2001; Tseng et al., 2001; Sharott et al., 2005; Dejean et al., 2008; Brazhnik et al., 2012). However, the relative contributions of altered cortico-BG-thalamocortical activity, loss of direct dopaminergic input to the cortex, or changes in wider networks in causing these cortical changes remains unclear.

Despite this evidence for widespread disruption of activity in structures afferent to Mthal, in the few studies performed to date, Mthal activity was not dramatically changed after dopamine depletion in animal models of PD. We might expect that Mthal neurons have lower firing rates, display bursty and oscillatory activity and exhibit LTS bursts for prolonged periods of time in PD, due to the increased activity of the output nuclei of the BG, and hence inhibitory tone in Mthal (Albin et al., 1989; Alexander and Crutcher, 1990; DeLong, 1990). One study in the awake monkey reports less specificity in receptive fields (Pessiglione et al., 2005), which is similar to reports in BG and cortex (Filion et al., 1988; Bergman et al., 1994; Abosch et al., 2002; Goldberg et al., 2002; Guehl et al., 2003). Other studies in anesthetized cats found decreases in Mthal firing rate (Voloshin et al., 1994; Schneider and Rothblat, 1996). These changes in Mthal activity are relatively mild, given the profound changes in neuronal activity in BG output nuclei. At present, there is no consensus on the effect of dopamine depletion on LTS bursts; of two studies examining LTS burst occurrence in the Mthal of PD patients one reported very few neurons exhibiting LTS bursts (Zirh et al., 1998), whereas the other found a high occurrence of LTS bursts (Magnin et al., 2000) and this discrepancy is probably due to the different parameters used to detect LTS bursts. To date, no animal studies have addressed changes or role of LTS bursts in Mthal in PD.

Data recorded from patients during surgery to implant electrodes to treat motor symptoms, such as tremor or rigidity provide useful information about the Mthal but they do not enable a direct comparison between control and PD patients; typically data from PD patients are compared to patients with another neurological disorder such as essential tremor or multiple sclerosis (Zirh et al., 1998; Raeva et al., 1998; Magnin et al., 2000; Brodkey et al., 2004; Hanson et al., 2012). A major finding of these human studies is that oscillatory signals occurring mainly in VLp neurons are coherent with the 3–8 Hz tremor and in synchrony with other Mthal neurons (Lenz et al., 1985, 1988, 1994; Zirh et al., 1998; Magnin et al., 2000; Marsden et al., 2000; Brodkey et al., 2004; Hanson et al., 2012). The origin of this oscillatory pattern of activity remains unknown, in particular, it has not been established whether the oscillatory activity is a cause or a consequence of the tremor. One hypothesis is that tremor arises from aberrant re-afferentation from cerebellar pathways to VLp and this pathway is normally used for rapid voluntary movements (Volkmann et al., 1996). Another theory suggests that the cerebellothalamocortical network produces the signal underlying the tremor and the BG network triggers when tremor occurs (Helmich et al., 2012). Because the BG and the cerebellum do not converge directly in the Mthal, this transfer presumably involves the cortex (Helmich et al., 2012).

Mthal lesions have been used for almost sixty years to treat tremor in PD (Hassler and Riechert, 1955). In recent years deep brain stimulation (DBS) of the thalamus has superseded thalamotomy primarily because side effects can be reduced by changing the stimulation parameters or stopped altogether by turning the stimulator off (Benabid et al., 1991; Koller et al., 1999; Okun and Vitek, 2004). Comparison of thalamotomy and DBS effects across studies is difficult, even when MRI has been used to determine anatomical landmarks, because of the inconsistent nomenclatures and subtleties in the placement of nuclei boundaries used for the human thalamus between research groups (Okun and Vitek, 2004) and difficulty visualizing some nuclei using MRI (Marsden et al., 2000; Bardinet et al., 2011). DBS has the advantage that the electrodes can cover a large area of thalamic volume and postsurgical testing can determine the best leads for effective stimulation (Katayama et al., 2005). Thalamotomy and DBS are highly effective for reducing the amplitude of tremor (Beuter and Titcombe, 2003; Duval et al., 2006; Mure et al., 2011). The position of the thalamotomy is critical, rather than the size of the lesion, and correlates with the degree of improvement (Atkinson et al., 2002). Notably, VLp lesion effectively treats tremor (Markham et al., 1966; Atkinson et al., 2002; Okun and Vitek, 2004; Klein et al., 2012). Similarly, high frequency (100–150 Hz) DBS within VLp (and to a lesser extent, VLa) achieved the best improvement in tremor (Yamamoto et al., 2004; Katayama et al., 2005; Klein et al., 2012). Clinical improvement of parkinsonian tremor occurs within 2–4 weeks of thalamotomy or DBS surgery and remains for 5–10 years without lasting side effects (Kelly and Gillingham, 1980; Nagaseki et al., 1986; Pahwa et al., 2006). A strategy used in thalamotomy and DBS surgery to improve outcomes is to target VLp based on the presence of oscillatory neuronal activity in the tremor range (Lenz et al., 1995; Garonzik et al., 2002). VLp thalamotomy and DBS are also effective for treating rigidity and quality of life (Markham et al., 1966; Benabid et al., 1991; Atkinson et al., 2002; Okun and Vitek, 2004; Klein et al., 2012). In contrast, the effect of VLp thalamotomy or DBS on bradykinesia, akinesia and fine motor control remains unclear with some studies reporting improvements (Perret, 1968; Perret et al., 1970), and other studies reporting no changes (Markham et al., 1966; Benabid et al., 1991; Beuter and Titcombe, 2003; Duval et al., 2006) or deleterious effects (van Someren et al., 1993; Boecker et al., 1997). Although the pathophysiology underlying PD tremor remains unknown, thalamotomy and DBS treatments support the idea that the VLp preferentially propagates oscillatory signals associated with tremor. It is possible that neural signals in other regions of Mthal play a role in parkinsonian akinesia and bradykinesia, such as the VA and VLa (Bornschlegl and Asanuma, 1987; Canavan et al., 1989; Okun and Vitek, 2004).

There is accumulating evidence that Mthal oscillatory activity in beta (12–30 Hz) and gamma (30–100 Hz) ranges is altered in PD. In the cortex and BG of PD patients and animal models, beta and gamma range oscillatory activities are routinely recorded, and are altered following administration of dopaminergic drugs (Levy et al., 2000, 2002; Brown et al., 2001; Sharott et al., 2005; Marceglia et al., 2006; Weinberger et al., 2006; Kuhn et al., 2009; Avila et al., 2010; Giannicola et al., 2010; Jenkinson and Brown, 2011; Brazhnik et al., 2012; Jenkinson et al., 2013), which provides support that these signals are pathological in PD. Similarly, oscillatory activity in the beta and gamma ranges have been reported in the cerebellar territory of patients (Paradiso et al., 2004; Kempf et al., 2009; Holdefer et al., 2010; Hanson et al., 2012; Brucke et al., 2013), and may also be pathological because they decrease and increase, respectively, during movements (Paradiso et al., 2004; Brucke et al., 2013), and gamma activity increases following administration of dopaminergic medication (Kempf et al., 2009). These surgeries were performed to implant stimulating electrodes into the VLp to treat tremor, so we do not know if VM, VA or VLa also show changes in oscillatory activity in beta and gamma ranges. To better understand the pathophysiology of PD, we need to know if synchronized activity in the beta and gamma ranges in Mthal is caused by a specific property of Mthal neurons in only the cerebellar territory or arises from inputs from the BG, cortex and/or cerebellum.

Taken together, the available data suggest that in normal conditions Mthal receives unsynchronized and non-bursty afferent signals from the cortex and BG, and that in PD this is profoundly altered so that the Mthal receives highly synchronized, oscillatory inputs. To date, the impact of this change in afferent activity on Mthal does not appear to have been fully characterized. For example, an issue that needs to be explored during movements in PD patients or animals models is whether Mthal neurons display the bursty and oscillatory activity seen in the BG and cortex. Bursty activity in the Mthal could be expected by two ways. First, if the cortex was the main driver, a bursty input will cause a coincident burst of activity in Mthal neurons, without necessarily causing LTS bursts. Second, if we consider the BG as a main driver, the profoundly bursty input from BG could produce a long inhibition in the Mthal that evokes LTS bursts during the interburst period i.e., when BG output is silent. In addition, if we consider that the cortex, cerebellum and BG are drivers, the occurrence of bursts and LTS bursts in Mthal will mainly depend on the timing of these inputs. It remains unclear how cerebellar activity is altered in PD. However, because BG and cortex seem to be synchronized and in-phase in PD (Williams et al., 2002; Goldberg et al., 2004; Walters et al., 2007; Dejean et al., 2008; Brazhnik et al., 2012), bursts from the cortex could be cancelled out by the profoundly strong, synchronized bursts from the BG. If this is the case, the occurrence and intensity of LTS bursts would not be as great as predicted. Recently, it has been proposed that pathological BG activity produces noise in the Mthal that compromises the fidelity of the Mthal output to the cortex, notably when a spike from the cortex gives rise to an LTS burst in the Mthal, due to pathological BG input (Guo et al., 2008; Rubin et al., 2012).

## Conclusion and future directions

Mthal is a structure strategically situated between the BG, cerebellum and cortex, but how it processes information from these three structures remains to be determined. We have reviewed two current approaches to information processing in the Mthal. The first applies driver/modulator concepts from sensory thalamus to Mthal, and proposes that Mthal subregions are driven by one input and modulated by others. However, the data do not support such a simple functional distinction, and suggest instead the possibility that there may be multiple driver or driver-like inputs, including the possibility that BG may strongly influence activity in the BG territory of Mthal.

The second approach, derived mainly from considerations of the cortico-BG circuit treats the BG receiving Mthal as a conduit for BG activity by rebound, gating or entrainment mechanisms. To date, studies exploring these mechanisms have not considered the role of other inputs. The rebound mechanism proposes that BG trigger LTS bursts following a period of inhibition. The main drawback of this rebound model, however, is that LTS bursts seem to rarely occur in awake conditions. The gating model proposes that BG control Mthal activity by a disinhibiting process when the direct pathway of BG is activated. In this model, the BG may act in cooperation with the cortex to drive Mthal neurons. A more recent model, called the entrainment model, proposes that when Mthal and BG neurons have a very high firing rate, Mthal is driven by the cortex and BG play an essential role determining the time window in which thalamic spikes can occur.

Here, we propose a new approach that raises the possibility that BG inputs have a driver-like function in Mthal. Instead of treating Mthal as simply a relay structure, all inputs are important, with Mthal acting as an integrator of multiple inputs (each of which has already integrated multiple signals concerned with different aspects of motor control). In this view, Mthal emerges as a “super-integrator” of information from the cortex, the BG and the cerebellum. The cortex would initiate development of the motor programme, the cerebellar territory of the Mthal would process the complex proprioceptive information needed to produce an appropriate movement and the BG territory would process motivational information. All three pathways are necessary for motor learning and to evoke the optimal movement, and both Mthal territories send super-integrated signals back to the cortex (Figure 4). Furthermore, the open feedback loops involving the BG, cerebellum, Mthal and cortex ensure that motivational and proprioceptive aspects of the movement are incorporated into the highly integrated motor programme that develops in the Mthal.

This new approach to understanding Mthal function needs to apply across a range of behavioral states and pathophysiology. The Mthal shows modulations in firing rate with respect to movement, and although motor deficits are severe in most animal models of PD, the reported changes in Mthal activity are relatively minor. However, it is critical that future experiments investigating Mthal function are conducted in behaving animals with simultaneous recordings of Mthal, cortex, BG and cerebellum, to ensure that the processing of context relevant information is explored in the Mthal.

Many aspects of the super-integrator model require further investigation. In particular, to test the hypothesis it is necessary to elucidate the respective weights and roles of cortical, cerebellar and BG afferents on Mthal activity and how they interact to fine-tune Mthal activity. Moreover, it is essential to know the functionality of the BG-Mthal giant synapse in mammals and to determine how and when LTS bursts occur in the Mthal. Ultimately, understanding how the Mthal processes information and the role of cortico-BG and corticocerebellar loops will improve our understanding of how the brain controls movement and the mechanisms underlying movement disorders such as PD.

## Conflict of interest statement

The authors declare that the research was conducted in the absence of any commercial or financial relationships that could be construed as a potential conflict of interest.

## References

[B1] Abosch A. Hutchison W. D. Saint-Cyr J. A. Dostrovsky J. O. Lozano A. M. (2002). Movement-related neurons of the subthalamic nucleus in patients with Parkinson disease. J. Neurosurg. 97, 1167–1172 10.3171/jns.2002.97.5.1167 12450039

[B2] Akkal D. Dum R. P. Strick P. L. (2007). Supplementary motor area and presupplementary motor area: targets of basal ganglia and cerebellar output. J. Neurosci. 27, 10659–10673 10.1523/jneurosci.3134-07.2007 17913900 PMC6672811

[B3] Albin R. L. Young A. B. Penney J. B. (1989). The functional anatomy of basal ganglia disorders. Trends Neurosci. 12, 366–375 10.1016/0166-2236(89)90074-X 2479133

[B4] Alexander G. E. Crutcher M. D. (1990). Functional architecture of basal ganglia circuits: neural substrates of parallel processing. Trends Neurosci. 13, 266–271 10.1016/0166-2236(90)90107-l 1695401

[B5] Anderson M. E. Devito J. L. (1987). An analysis of potentially converging inputs to the rostral ventral thalamic nuclei of the cat. Exp. Brain Res. 68, 260–276 10.1007/bf00248792 3691701

[B6] Anderson M. E. Turner R. S. (1991). Activity of neurons in cerebellar-receiving and pallidal-receiving areas of the thalamus of the behaving monkey. J. Neurophysiol. 66, 879–893 1753292 10.1152/jn.1991.66.3.879

[B7] Anderson M. E. Yoshida M. (1980). Axonal branching patterns and location of nigrothalamic and nigrocollicular neurons in the cat. J. Neurophysiol. 43, 883–895 7359179 10.1152/jn.1980.43.4.883

[B8] Arai R. Jacobowitz D. M. Deura S. (1994). Distribution of calretinin, calbindin-D28k, and parvalbumin in the rat thalamus. Brain Res. Bull. 33, 595–614 10.1016/0361-9230(94)90086-8 8187003

[B9] Atherton J. F. Bevan M. D. (2005). Ionic mechanisms underlying autonomous action potential generation in the somata and dendrites of GABAergic substantia nigra pars reticulata neurons in vitro. J. Neurosci. 25, 8272–8281 10.1523/jneurosci.1475-05.2005 16148235 PMC6725542

[B10] Atkinson J. D. Collins D. L. Bertrand G. Peters T. M. Pike G. B. Sadikot A. F. (2002). Optimal location of thalamotomy lesions for tremor associated with Parkinson disease: a probabilistic analysis based on postoperative magnetic resonance imaging and an integrated digital atlas. J. Neurosurg. 96, 854–866 10.3171/jns.2002.96.5.0854 12005392

[B11] Aumann T. D. Rawson J. A. Finkelstein D. I. Horne M. K. (1994). Projections from the lateral and interposed cerebellar nuclei to the thalamus of the rat: a light and electron microscopic study using single and double anterograde labelling. J. Comp. Neurol. 349, 165–181 10.1002/cne.903490202 7860776

[B12] Avila I. Parr-Brownlie L. C. Brazhnik E. Castaneda E. Bergstrom D. A. Walters J. R. (2010). Beta frequency synchronization in basal ganglia output during rest and walk in a hemiparkinsonian rat. Exp. Neurol. 221, 307–319 10.1016/j.expneurol.2009.11.016 19948166 PMC3384738

[B13] Baker S. N. Olivier E. Lemon R. N. (1997). Coherent oscillations in monkey motor cortex and hand muscle EMG show task-dependent modulation. J. Physiol. 501, ( Pt 1) 225–241 10.1111/j.1469-7793.1997.225bo.x 9175005 PMC1159515

[B14] Baker S. N. Spinks R. Jackson A. Lemon R. N. (2001). Synchronization in monkey motor cortex during a precision grip task. I. Task-dependent modulation in single-unit synchrony. J. Neurophysiol. 85, 869–885 11160519 10.1152/jn.2001.85.2.869

[B15] Bardinet E. Belaid H. Grabli D. Welter M. L. Vidal S. F. Galanaud D. (2011). Thalamic stimulation for tremor: can target determination be improved? Mov. Disord. 26, 307–312 10.1002/mds.23448 21412838

[B16] Bar-Gad I. Morris G. Bergman H. (2003). Information processing, dimensionality reduction and reinforcement learning in the basal ganglia. Prog. Neurobiol. 71, 439–473 10.1016/j.pneurobio.2003.12.001 15013228

[B17] Barnes T. D. Kubota Y. Hu D. Jin D. Z. Graybiel A. M. (2005). Activity of striatal neurons reflects dynamic encoding and recoding of procedural memories. Nature 437, 1158–1161 10.1038/nature04053 16237445

[B18] Barth A. L. Poulet J. F. (2012). Experimental evidence for sparse firing in the neocortex. Trends Neurosci. 35, 345–355 10.1016/j.tins.2012.03.008 22579264

[B19] Baufreton J. Atherton J. F. Surmeier D. J. Bevan M. D. (2005). Enhancement of excitatory synaptic integration by GABAergic inhibition in the subthalamic nucleus. J. Neurosci. 25, 8505–8517 10.1523/jneurosci.1163-05.2005 16162932 PMC6725678

[B20] Baunez C. Nieoullon A. Amalric M. (1995). In a rat model of parkinsonism, lesions of the subthalamic nucleus reverse increases of reaction time but induce a dramatic premature responding deficit. J. Neurosci. 15, 6531–6541 7472415 10.1523/JNEUROSCI.15-10-06531.1995 PMC6578020

[B21] Beloozerova I. N. Sirota M. G. Swadlow H. A. (2003a). Activity of different classes of neurons of the motor cortex during locomotion. J. Neurosci. 23, 1087–1097 12574439 10.1523/JNEUROSCI.23-03-01087.2003 PMC6741911

[B22] Beloozerova I. N. Sirota M. G. Swadlow H. A. Orlovsky G. N. Popova L. B. Deliagina T. G. (2003b). Activity of different classes of neurons of the motor cortex during postural corrections. J. Neurosci. 23, 7844–7853 12944514 10.1523/JNEUROSCI.23-21-07844.2003 PMC6740594

[B23] Benabid A. L. Pollak P. Gervason C. Hoffmann D. Gao D. M. Hommel M. (1991). Long-term suppression of tremor by chronic stimulation of the ventral intermediate thalamic nucleus. Lancet 337, 403–406 10.1016/0140-6736(91)91175-t 1671433

[B24] Bergman H. Wichmann T. Karmon B. DeLong M. R. (1994). The primate subthalamic nucleus. II. Neuronal activity in the MPTP model of parkinsonism. J. Neurophysiol. 72, 507–520 7983515 10.1152/jn.1994.72.2.507

[B25] Beuter A. Titcombe M. S. (2003). Modulation of tremor amplitude during deep brain stimulation at different frequencies. Brain Cogn. 53, 190–192 10.1016/s0278-2626(03)00107-6 14607145

[B26] Bezdudnaya T. Cano M. Bereshpolova Y. Stoelzel C. R. Alonso J. M. Swadlow H. A. (2006). Thalamic burst mode and inattention in the awake LGNd. Neuron 49, 421–432 10.1016/j.neuron.2006.01.010 16446145

[B27] Bodor A. L. Giber K. Rovo Z. Ulbert I. Acsady L. (2008). Structural correlates of efficient GABAergic transmission in the basal ganglia-thalamus pathway. J. Neurosci. 28, 3090–3102 10.1523/jneurosci.5266-07.2008 18354012 PMC2670451

[B28] Boecker H. Wills A. J. Ceballos-Baumann A. Samuel M. Thomas D. G. Marsden C. D. (1997). Stereotactic thalamotomy in tremor-dominant Parkinson’s disease: an H2(15)O PET motor activation study. Ann. Neurol. 41, 108–111 9005873 10.1002/ana.410410118

[B29] Boraud T. Bezard E. Bioulac B. Gross C. E. (2002). From single extracellular unit recording in experimental and human Parkinsonism to the development of a functional concept of the role played by the basal ganglia in motor control. Prog. Neurobiol. 66, 265–283 10.1016/s0301-0082(01)00033-8 11960681

[B30] Bornschlegl M. Asanuma H. (1987). Importance of the projection from the sensory to the motor cortex for recovery of motor function following partial thalamic lesion in the monkey. Brain Res. 437, 121–130 10.1016/0006-8993(87)91533-2 2827861

[B31] Borst J. G. (2010). The low synaptic release probability in vivo. Trends Neurosci. 33, 259–266 10.1016/j.tins.2010.03.003 20371122

[B32] Bosch C. Degos B. Deniau J. M. Venance L. (2011). Subthalamic nucleus high-frequency stimulation generates a concomitant synaptic excitation-inhibition in substantia nigra pars reticulata. J. Physiol. 589, 4189–4207 10.1113/jphysiol.2011.211367 21690190 PMC3180578

[B33] Braitenberg V. Heck D. Sultan F. (1997). The detection and generation of sequences as a key to cerebellar function: experiments and theory. Behav. Brain Sci. 20, 229–245 discussion 245–277. 10.1017/s0140525x9721143x 10096998

[B34] Brazhnik E. Cruz A. V. Avila I. Wahba M. I. Novikov N. Ilieva N. M. (2012). State-dependent spike and local field synchronization between motor cortex and substantia nigra in hemiparkinsonian rats. J. Neurosci. 32, 7869–7880 10.1523/jneurosci.0943-12.2012 22674263 PMC3423905

[B35] Brodkey J. A. Tasker R. R. Hamani C. Mcandrews M. P. Dostrovsky J. O. Lozano A. M. (2004). Tremor cells in the human thalamus: differences among neurological disorders. J. Neurosurg. 101, 43–47 10.3171/jns.2004.101.1.0043 15255250

[B36] Brown P. Oliviero A. Mazzone P. Insola A. Tonali P. Di Lazzaro V. (2001). Dopamine dependency of oscillations between subthalamic nucleus and pallidum in Parkinson’s disease. J. Neurosci. 21, 1033–1038 11157088 10.1523/JNEUROSCI.21-03-01033.2001 PMC6762327

[B37] Brucke C. Bock A. Huebl J. Krauss J. K. Schonecker T. Schneider G. H. (2013). Thalamic gamma oscillations correlate with reaction time in a Go/noGo task in patients with essential tremor. Neuroimage 75, 36–45 10.1016/j.neuroimage.2013.02.038 23466935

[B38] Butler E. G. Finkelstein D. I. Harvey M. C. Churchward P. R. Forlano L. M. Horne M. K. (1996). The relationship between monkey ventrolateral thalamic nucleus activity and kinematic parameters of wrist movement. Brain Res. 736, 146–159 10.1016/s0006-8993(96)00672-5 8930319

[B39] Butler E. G. Horne M. K. Hawkins N. J. (1992). The activity of monkey thalamic and motor cortical neurones in a skilled, ballistic movement. J. Physiol. 445, 25–48 1501135 10.1113/jphysiol.1992.sp018910 PMC1179968

[B40] Caminiti R. Johnson P. B. Urbano A. (1990). Making arm movements within different parts of space: dynamic aspects in the primate motor cortex. J. Neurosci. 10, 2039–2058 2376768 10.1523/JNEUROSCI.10-07-02039.1990 PMC6570378

[B41] Canavan A. G. Nixon P. D. Passingham R. E. (1989). Motor learning in monkeys (Macaca fascicularis) with lesions in motor thalamus. Exp. Brain Res. 77, 113–126 10.1007/bf00250573 2792254

[B42] Chen W. Zhang J. J. Hu G. Y. Wu C. P. (1996). Electrophysiological and morphological properties of pyramidal and nonpyramidal neurons in the cat motor cortex in vitro. Neuroscience 73, 39–55 10.1016/0306-4522(96)00009-7 8783228

[B43] Chevalier G. Deniau J. M. (1982). Inhibitory nigral influence on cerebellar evoked responses in the rat ventromedial thalamic nucleus. Exp. Brain Res. 48, 369–376 10.1007/bf00238613 7151930

[B44] Chevalier G. Deniau J. M. (1990). Disinhibition as a basic process in the expression of striatal functions. Trends Neurosci. 13, 277–280 10.1016/0166-2236(90)90109-n 1695403

[B45] Churchland M. M. Cunningham J. P. Kaufman M. T. Foster J. D. Nuyujukian P. Ryu S. I. (2012). Neural population dynamics during reaching. Nature 487, 51–56 10.1038/nature11129 22722855 PMC3393826

[B46] Connelly W. M. Errington A. C. (2012). Temporally selective firing of cortical and thalamic neurons during sleep and wakefulness. J. Neurosci. 32, 7415–7417 10.1523/jneurosci.1164-12.2012 22649221 PMC3382954

[B47] Crick F. (1984). Function of the thalamic reticular complex: the searchlight hypothesis. Proc. Natl. Acad. Sci. U S A 81, 4586–4590 10.1073/pnas.81.14.4586 6589612 PMC345636

[B48] Crunelli V. Hughes S. W. (2010). The slow (<1 Hz) rhythm of non-REM sleep: a dialogue between three cardinal oscillators. Nat. Neurosci. 13, 9–17 10.1038/nn.2445 19966841 PMC2980822

[B49] D’Angelo E. Mazzarello P. Prestori F. Mapelli J. Solinas S. Lombardo P. (2011). The cerebellar network: from structure to function and dynamics. Brain Res. Rev. 66, 5–15 10.1016/j.brainresrev.2010.10.002 20950649

[B50] Dean P. Porrill J. Ekerot C. F. Jorntell H. (2010). The cerebellar microcircuit as an adaptive filter: experimental and computational evidence. Nat. Rev. Neurosci. 11, 30–43 10.1038/nrn2756 19997115

[B51] Dejean C. Gross C. E. Bioulac B. Boraud T. (2008). Dynamic changes in the cortex-basal ganglia network after dopamine depletion in the rat. J. Neurophysiol. 100, 385–396 10.1152/jn.90466.2008 18497362

[B52] DeLong M. R. (1971). Activity of pallidal neurons during movement. J. Neurophysiol. 34, 414–427 4997823 10.1152/jn.1971.34.3.414

[B53] DeLong M. R. (1990). Primate models of movement disorders of basal ganglia origin. Trends Neurosci. 13, 281–285 10.1016/0166-2236(90)90110-v 1695404

[B54] Deniau J. M. Chevalier G. (1985). Disinhibition as a basic process in the expression of striatal functions. II. The striato-nigral influence on thalamocortical cells of the ventromedial thalamic nucleus. Brain Res. 334, 227–233 3995318 10.1016/0006-8993(85)90214-8

[B55] Deniau J. M. Lackner D. Feger J. (1978). Effect of substantia nigra stimulation on identified neurons in the VL-VA thalamic complex: comparison between intact and chronically decorticated cats. Brain Res. 145, 27–35 10.1016/0006-8993(78)90793-x 638780

[B56] Di Chiara G. Morelli M. Porceddu M. L. Gessa G. L. (1979). Role of thalamic gamma-aminobutyrate in motor functions: catalepsy and ipsiversive turning after intrathalamic muscimol. Neuroscience 4, 1453–1465 10.1016/0306-4522(79)90050-2 575998

[B57] Doupe A. J. Perkel D. J. Reiner A. Stern E. A. (2005). Birdbrains could teach basal ganglia research a new song. Trends Neurosci. 28, 353–363 10.1016/j.tins.2005.05.005 15935486

[B58] Duval C. Panisset M. Strafella A. P. Sadikot A. F. (2006). The impact of ventrolateral thalamotomy on tremor and voluntary motor behavior in patients with Parkinson’s disease. Exp. Brain Res. 170, 160–171 10.1007/s00221-005-0198-4 16328283

[B59] Ebner T. J. Hewitt A. L. Popa L. S. (2011). What features of limb movements are encoded in the discharge of cerebellar neurons? Cerebellum 10, 683–693 10.1007/s12311-010-0243-0 21203875 PMC3711690

[B60] Eccles J. C. (1973). The cerebellum as a computer: patterns in space and time. J Physiol. 229, 1–32 4347742 10.1113/jphysiol.1973.sp010123 PMC1350208

[B61] Fan D. Rossi M. A. Yin H. H. (2012). Mechanisms of action selection and timing in substantia nigra neurons. J. Neurosci. 32, 5534–5548 10.1523/jneurosci.5924-11.2012 22514315 PMC6703499

[B62] Fang P. C. Stepniewska I. Kaas J. H. (2006). The thalamic connections of motor, premotor, and prefrontal areas of cortex in a prosimian primate (Otolemur garnetti). Neuroscience 143, 987–1020 10.1016/j.neuroscience.2006.08.053 17055664 PMC1832073

[B63] Farrant M. Nusser Z. (2005). Variations on an inhibitory theme: phasic and tonic activation of GABA(A) receptors. Nat. Rev. Neurosci. 6, 215–229 10.1038/nrn1625 15738957

[B64] Filion M. Tremblay L. Bedard P. J. (1988). Abnormal influences of passive limb movement on the activity of globus pallidus neurons in parkinsonian monkeys. Brain Res. 444, 165–176 10.1016/0006-8993(88)90924-9 3359286

[B65] Forlano L. M. Horne M. K. Butler E. G. Finkelstein D. (1993). Neural activity in the monkey anterior ventrolateral thalamus during trained, ballistic movements. J. Neurophysiol. 70, 2276–2288 8120582 10.1152/jn.1993.70.6.2276

[B66] Franks N. P. (2008). General anaesthesia: from molecular targets to neuronal pathways of sleep and arousal. Nat. Rev. Neurosci. 9, 370–386 10.1038/nrn2372 18425091

[B67] Fujimoto K. Kita H. (1992). Responses of rat substantia nigra pars reticulata units to cortical stimulation. Neurosci. Lett. 142, 105–109 10.1016/0304-3940(92)90630-p 1407709

[B68] Garonzik I. M. Hua S. E. Ohara S. Lenz F. A. (2002). Intraoperative microelectrode and semi-microelectrode recording during the physiological localization of the thalamic nucleus ventral intermediate. Mov. Disord. 17 Suppl. 3, S135–S144 10.1002/mds.10155 11948768

[B69] Georgopoulos A. P. (1988). Neural integration of movement: role of motor cortex in reaching. FASEB J. 2, 2849–2857 3139485 10.1096/fasebj.2.13.3139485

[B70] Georgopoulos A. P. DeLong M. R. Crutcher M. D. (1983). Relations between parameters of step-tracking movements and single cell discharge in the globus pallidus and subthalamic nucleus of the behaving monkey. J. Neurosci. 3, 1586–1598 6875658 10.1523/JNEUROSCI.03-08-01586.1983 PMC6564524

[B71] Georgopoulos A. P. Kalaska J. F. Caminiti R. Massey J. T. (1982). On the relations between the direction of two-dimensional arm movements and cell discharge in primate motor cortex. J. Neurosci. 2, 1527–1537 7143039 10.1523/JNEUROSCI.02-11-01527.1982 PMC6564361

[B72] Georgopoulos A. P. Schwartz A. B. Kettner R. E. (1986). Neuronal population coding of movement direction. Science 233, 1416–1419 10.1126/science.3749885 3749885

[B73] Giannicola G. Marceglia S. Rossi L. Mrakic-Sposta S. Rampini P. Tamma F. (2010). The effects of levodopa and ongoing deep brain stimulation on subthalamic beta oscillations in Parkinson’s disease. Exp. Neurol. 226, 120–127 10.1016/j.expneurol.2010.08.011 20713047

[B74] Goldberg J. A. Boraud T. Maraton S. Haber S. N. Vaadia E. Bergman H. (2002). Enhanced synchrony among primary motor cortex neurons in the 1-methyl-4-phenyl-1,2,3,6-tetrahydropyridine primate model of Parkinson’s disease. J. Neurosci. 22, 4639–4653 12040070 10.1523/JNEUROSCI.22-11-04639.2002 PMC6758785

[B76] Goldberg J. H. Farries M. A. Fee M. S. (2012). Integration of cortical and pallidal inputs in the basal ganglia-recipient thalamus of singing birds. J. Neurophysiol. 108, 1403–1429 10.1152/jn.00056.2012 22673333 PMC3544964

[B77] Goldberg J. H. Fee M. S. (2011). Vocal babbling in songbirds requires the basal ganglia-recipient motor thalamus but not the basal ganglia. J. Neurophysiol. 105, 2729–2739 10.1152/jn.00823.2010 21430276 PMC3118735

[B78] Goldberg J. H. Fee M. S. (2012). A cortical motor nucleus drives the basal ganglia-recipient thalamus in singing birds. Nat. Neurosci. 15, 620–627 10.1038/nn.3047 22327474 PMC3321369

[B75] Goldberg J. A. Rokni U. Boraud T. Vaadia E. Bergman H. (2004). Spike synchronization in the cortex/basal-ganglia networks of Parkinsonian primates reflects global dynamics of the local field potentials. J. Neurosci. 24, 6003–6010 10.1523/jneurosci.4848-03.2004 15229247 PMC6729228

[B79] Grofova I. Rinvik E. (1974). Cortical and pallidal projections to the nucleus ventralis lateralis thalami. Electron microscopical studies in the cat. Anat. Embryol. (Berl) 146, 113–132 10.1007/bf00315589 4618726

[B80] Guehl D. Pessiglione M. Francois C. Yelnik J. Hirsch E. C. Feger J. (2003). Tremor-related activity of neurons in the ‘motor’ thalamus: changes in firing rate and pattern in the MPTP vervet model of parkinsonism. Eur. J. Neurosci. 17, 2388–2400 10.1046/j.1460-9568.2003.02685.x 12814370

[B81] Gulcebi M. I. Ketenci S. Linke R. Hacioglu H. Yanali H. Veliskova J. (2012). Topographical connections of the substantia nigra pars reticulata to higher-order thalamic nuclei in the rat. Brain Res. Bull. 87, 312–318 10.1016/j.brainresbull.2011.11.005 22108631

[B82] Gulledge A. T. Kampa B. M. Stuart G. J. (2005). Synaptic integration in dendritic trees. J. Neurobiol. 64, 75–90 10.1002/neu.20207 15884003

[B83] Guo Y. Rubin J. E. Mcintyre C. C. Vitek J. L. Terman D. (2008). Thalamocortical relay fidelity varies across subthalamic nucleus deep brain stimulation protocols in a data-driven computational model. J. Neurophysiol. 99, 1477–1492 10.1152/jn.01080.2007 18171706

[B84] Haber S. N. Calzavara R. (2009). The cortico-basal ganglia integrative network: the role of the thalamus. Brain Res. Bull. 78, 69–74 10.1016/j.brainresbull.2008.09.013 18950692 PMC4459637

[B85] Hanson T. L. Fuller A. M. Lebedev M. A. Turner D. A. Nicolelis M. A. (2012). Subcortical neuronal ensembles: an analysis of motor task association, tremor, oscillations, and synchrony in human patients. J. Neurosci. 32, 8620–8632 10.1523/jneurosci.0750-12.2012 22723703 PMC3502028

[B86] Hassani O. K. Mouroux M. Feger J. (1996). Increased subthalamic neuronal activity after nigral dopaminergic lesion independent of disinhibition via the globus pallidus. Neuroscience 72, 105–115 10.1016/0306-4522(95)00535-8 8730710

[B87] Hassler R. (1978). Striatal control of locomotion, intentional actions and of integrating and perceptive activity. J. Neurol. Sci. 36, 187–224 10.1016/0022-510x(78)90082-5 206673

[B88] Hassler R. Riechert T. (1955). A special method of stereotactic brain operation. Proc. R. Soc. Med. 48, 469–470 14395256 10.1177/003591575504800620 PMC1919062

[B89] Hazrati L. N. Parent A. (1991). Contralateral pallidothalamic and pallidotegmental projections in primates: an anterograde and retrograde labeling study. Brain Res. 567, 212–223 10.1016/0006-8993(91)90798-z 1817727

[B90] Helmich R. C. Hallett M. Deuschl G. Toni I. Bloem B. R. (2012). Cerebral causes and consequences of parkinsonian resting tremor: a tale of two circuits? Brain 135, 3206–3226 10.1093/brain/aws023 22382359 PMC3501966

[B91] Hikosaka O. (2007). GABAergic output of the basal ganglia. Prog. Brain Res. 160, 209–226 10.1016/s0079-6123(06)60012-5 17499116

[B92] Hirai T. Jones E. G. (1989). A new parcellation of the human thalamus on the basis of histochemical staining. Brain Res. Brain Res. Rev. 14, 1–34 10.1016/0165-0173(89)90007-6 2720229

[B93] Hirsch J. C. Fourment A. Marc M. E. (1983). Sleep-related variations of membrane potential in the lateral geniculate body relay neurons of the cat. Brain Res. 259, 308–312 10.1016/0006-8993(83)91264-7 6297675

[B94] Holdefer R. N. Cohen B. A. Greene K. A. (2010). Intraoperative local field recording for deep brain stimulation in Parkinson’s disease and essential tremor. Mov. Disord. 25, 2067–2075 10.1002/mds.23232 20721922

[B95] Hooks B. M. Mao T. Gutnisky D. A. Yamawaki N. Svoboda K. Shepherd G. M. (2013). Organization of cortical and thalamic input to pyramidal neurons in mouse motor cortex. J. Neurosci. 33, 748–760 10.1523/jneurosci.4338-12.2013 23303952 PMC3710148

[B96] Hoover J. E. Strick P. L. (1993). Multiple output channels in the basal ganglia. Science 259, 819–821 10.1126/science.7679223 7679223

[B97] Horak F. B. Anderson M. E. (1984). Influence of globus pallidus on arm movements in monkeys. II. Effects of stimulation. J. Neurophysiol. 52, 305–322 6481435 10.1152/jn.1984.52.2.305

[B98] Horne M. K. Butler E. G. (1995). The role of the cerebello-thalamo-cortical pathway in skilled movement. Prog. Neurobiol. 46, 199–213 10.1016/0301-0082(95)00002-d 7568913

[B99] Horne M. K. Porter R. (1980). The discharges during movement of cells in the ventrolateral thalamus of the conscious monkey. J. Physiol. 304, 349–372 7441539 10.1113/jphysiol.1980.sp013328 PMC1282934

[B100] Hsu C. L. Yang H. W. Yen C. T. Min M. Y. (2012). A requirement of low-threshold calcium spike for induction of spike-timing-dependent plasticity at corticothalamic synapses on relay neurons in the ventrobasal nucleus of rat thalamus. Chin. J. Physiol. 55, 380–389 10.4077/cjp.2012.baa047 23286445

[B101] Hubel D. H. Wiesel T. N. (1961). Integrative action in the cat’s lateral geniculate body. J. Physiol. 155, 385–398 13716436 10.1113/jphysiol.1961.sp006635 PMC1359861

[B102] Huguenard J. R. McCormick D. A. (1992). Simulation of the currents involved in rhythmic oscillations in thalamic relay neurons. J. Neurophysiol. 68, 1373–1383 1279135 10.1152/jn.1992.68.4.1373

[B103] Hutchison W. D. Dostrovsky J. O. Walters J. R. Courtemanche R. Boraud T. Goldberg J. (2004). Neuronal oscillations in the basal ganglia and movement disorders: evidence from whole animal and human recordings. J. Neurosci. 24, 9240–9243 10.1523/jneurosci.3366-04.2004 15496658 PMC6730107

[B104] Inase M. Buford J. A. Anderson M. E. (1996). Changes in the control of arm position, movement, and thalamic discharge during local inactivation in the globus pallidus of the monkey. J. Neurophysiol. 75, 1087–1104 8867120 10.1152/jn.1996.75.3.1087

[B105] Isomura Y. Harukuni R. Takekawa T. Aizawa H. Fukai T. (2009). Microcircuitry coordination of cortical motor information in self-initiation of voluntary movements. Nat. Neurosci. 12, 1586–1593 10.1038/nn.2431 19898469

[B106] Ivanusic J. J. Bourke D. W. Xu Z. M. Butler E. G. Horne M. K. (2005). Cerebellar thalamic activity in the macaque monkey encodes the duration but not the force or velocity of wrist movement. Brain Res. 1041, 181–197 10.1016/j.brainres.2005.02.005 15829227

[B107] Jacobson G. A. Rokni D. Yarom Y. (2008). A model of the olivo-cerebellar system as a temporal pattern generator. Trends Neurosci. 31, 617–625 10.1016/j.tins.2008.09.005 18952303

[B108] Jaeger D. Gilman S. Aldridge J. W. (1995). Neuronal activity in the striatum and pallidum of primates related to the execution of externally cued reaching movements. Brain Res. 694, 111–127 10.1016/0006-8993(95)00780-t 8974634

[B109] Jahnsen H. Llinas R. (1984a). Electrophysiological properties of guinea-pig thalamic neurones: an in vitro study. J. Physiol. 349, 205–226 6737292 10.1113/jphysiol.1984.sp015153 PMC1199334

[B110] Jahnsen H. Llinas R. (1984b). Ionic basis for the electro-responsiveness and oscillatory properties of guinea-pig thalamic neurones in vitro. J. Physiol. 349, 227–247 6737293 10.1113/jphysiol.1984.sp015154 PMC1199335

[B111] Jeljeli M. Strazielle C. Caston J. Lalonde R. (2003). Effects of ventrolateral-ventromedial thalamic lesions on motor coordination and spatial orientation in rats. Neurosci. Res. 47, 309–316 10.1016/s0168-0102(03)00224-4 14568112

[B112] Jenkinson N. Brown P. (2011). New insights into the relationship between dopamine, beta oscillations and motor function. Trends Neurosci. 34, 611–618 10.1016/j.tins.2011.09.003 22018805

[B113] Jenkinson N. Kuhn A. A. Brown P. (2013). gamma oscillations in the human basal ganglia. Exp. Neurol. 245, 72–76 10.1016/j.expneurol.2012.07.005 22841500

[B114] Joffroy A. J. Lamarre Y. (1974). Single cell activity in the ventral lateral thalamus of the unanesthetized monkey. Exp. Neurol. 42, 1–16 10.1016/0014-4886(74)90002-8 4207724

[B115] Jog M. S. Kubota Y. Connolly C. I. Hillegaart V. Graybiel A. M. (1999). Building neural representations of habits. Science 286, 1745–1749 10.1126/science.286.5445.1745 10576743

[B116] Johnson F. Bottjer S. W. (1993). Induced cell death in a thalamic nucleus during a restricted period of zebra finch vocal development. J. Neurosci. 13, 2452–2462 8501517 10.1523/JNEUROSCI.13-06-02452.1993 PMC6576511

[B117] Jones E. G. (2007). The Thalamus. 2nd Edn. Cambridge: Cambridge University Press

[B118] Kakei S. Na J. Shinoda Y. (2001). Thalamic terminal morphology and distribution of single corticothalamic axons originating from layers 5 and 6 of the cat motor cortex. J. Comp. Neurol. 437, 170–185 10.1002/cne.1277 11494250

[B119] Kalaska J. F. Caminiti R. Georgopoulos A. P. (1983). Cortical mechanisms related to the direction of two-dimensional arm movements: relations in parietal area 5 and comparison with motor cortex. Exp. Brain Res. 51, 247–260 10.1007/bf00237200 6617794

[B120] Kalaska J. F. Cohen D. A. Hyde M. L. Prud’homme M. (1989). A comparison of movement direction-related versus load direction-related activity in primate motor cortex, using a two-dimensional reaching task. J. Neurosci. 9, 2080–2102 2723767 10.1523/JNEUROSCI.09-06-02080.1989 PMC6569743

[B121] Katayama Y. Kano T. Kobayashi K. Oshima H. Fukaya C. Yamamoto T. (2005). Difference in surgical strategies between thalamotomy and thalamic deep brain stimulation for tremor control. J. Neurol. 252 Suppl. 4, IV17–IV22 10.1007/s00415-005-4005-8 16222433

[B122] Kelly P. J. Gillingham F. J. (1980). The long-term results of stereotaxic surgery and L-dopa therapy in patients with Parkinson’s disease. A 10-year follow-up study. J. Neurosurg. 53, 332–337 10.3171/jns.1980.53.3.0332 6999132

[B123] Kempf F. Brucke C. Salih F. Trottenberg T. Kupsch A. Schneider G. H. (2009). Gamma activity and reactivity in human thalamic local field potentials. Eur. J. Neurosci. 29, 943–953 10.1111/j.1460-9568.2009.06655.x 19291224

[B124] Kim J. S. Kim W. B. Kim Y. B. Lee Y. Kim Y. S. Shen F. Y. (2011). Chronic hyperosmotic stress converts GABAergic inhibition into excitation in vasopressin and oxytocin neurons in the rat. J. Neurosci. 31, 13312–13322 10.1523/jneurosci.1440-11.2011 21917814 PMC6623275

[B125] Klein J. C. Barbe M. T. Seifried C. Baudrexel S. Runge M. Maarouf M. (2012). The tremor network targeted by successful VIM deep brain stimulation in humans. Neurology 78, 787–795 10.1212/01.wnl.0000419345.94406.07 22377809

[B126] Klockgether T. Schwarz M. Turski L. Sontag K. H. (1986a). The rat ventromedial thalamic nucleus and motor control: role of N-methyl-D-aspartate-mediated excitation, GABAergic inhibition, and muscarinic transmission. J. Neurosci. 6, 1702–1711 2872282 10.1523/JNEUROSCI.06-06-01702.1986 PMC6568707

[B127] Klockgether T. Turski L. Schwarz M. Sontag K. H. (1986b). Motor actions of excitatory amino acids and their antagonists within the rat ventromedial thalamic nucleus. Brain Res. 399, 1–9 10.1016/0006-8993(86)90594-9 3026571

[B128] Klug A. Borst J. G. Carlson B. A. Kopp-Scheinpflug C. Klyachko V. A. Xu-Friedman M. A. (2012). How do short-term changes at synapses fine-tune information processing? J. Neurosci. 32, 14058–14063 10.1523/jneurosci.3348-12.2012 23055473 PMC3488594

[B129] Kojima S. Doupe A. J. (2009). Activity propagation in an avian basal ganglia-thalamocortical circuit essential for vocal learning. J. Neurosci. 29, 4782–4793 10.1523/jneurosci.4903-08.2009 19369547 PMC2685169

[B130] Koller W. C. Pahwa R. Lyons K. E. Albanese A. (1999). Surgical treatment of Parkinson’s disease. J. Neurol. Sci. 167, 1–10 10500254 10.1016/s0022-510x(99)00139-2

[B131] Kolomiets B. P. Deniau J. M. Glowinski J. Thierry A. M. (2003). Basal ganglia and processing of cortical information: functional interactions between trans-striatal and trans-subthalamic circuits in the substantia nigra pars reticulata. Neuroscience 117, 931–938 10.1016/s0306-4522(02)00824-2 12654344

[B132] Krack P. Dostrovsky J. Ilinsky I. Kultas-Ilinsky K. Lenz F. Lozano A. (2002). Surgery of the motor thalamus: problems with the present nomenclatures. Mov. Disord. 17 Suppl. 3, S2–S8 10.1002/mds.10136 11948749

[B133] Kuhn A. A. Tsui A. Aziz T. Ray N. Brucke C. Kupsch A. (2009). Pathological synchronisation in the subthalamic nucleus of patients with Parkinson’s disease relates to both bradykinesia and rigidity. Exp. Neurol. 215, 380–387 10.1016/j.expneurol.2008.11.008 19070616

[B134] Kultas-Ilinsky K. Ilinsky I. A. (1990). Fine structure of the magnocellular subdivision of the ventral anterior thalamic nucleus (VAmc) of Macaca mulatta: II. Organization of nigrothalamic afferents as revealed with EM autoradiography. J. Comp. Neurol. 294, 479–489 10.1002/cne.902940314 2341622

[B135] Kultas-Ilinsky K. Ilinsky I. A. (1991). Fine structure of the ventral lateral nucleus (VL) of the Macaca mulatta thalamus: cell types and synaptology. J. Comp. Neurol. 314, 319–349 10.1002/cne.903140209 1723998

[B136] Kultas-Ilinsky K. Sivan-Loukianova E. Ilinsky I. A. (2003). Reevaluation of the primary motor cortex connections with the thalamus in primates. J. Comp. Neurol. 457, 133–158 10.1002/cne.10539 12541315

[B137] Kuramoto E. Fujiyama F. Nakamura K. C. Tanaka Y. Hioki H. Kaneko T. (2011). Complementary distribution of glutamatergic cerebellar and GABAergic basal ganglia afferents to the rat motor thalamic nuclei. Eur. J. Neurosci. 33, 95–109 10.1111/j.1460-9568.2010.07481.x 21073550

[B138] Kuramoto E. Furuta T. Nakamura K. C. Unzai T. Hioki H. Kaneko T. (2009). Two types of thalamocortical projections from the motor thalamic nuclei of the rat: a single neuron-tracing study using viral vectors. Cereb. Cortex 19, 2065–2077 10.1093/cercor/bhn231 19174446

[B139] Kurata K. (2005). Activity properties and location of neurons in the motor thalamus that project to the cortical motor areas in monkeys. J. Neurophysiol. 94, 550–566 10.1152/jn.01034.2004 15703228

[B140] Leblois A. Bodor A. L. Person A. L. Perkel D. J. (2009). Millisecond timescale disinhibition mediates fast information transmission through an avian basal ganglia loop. J. Neurosci. 29, 15420–15433 10.1523/jneurosci.3060-09.2009 20007467 PMC2819911

[B141] Lemaire N. Hernandez L. F. Hu D. Kubota Y. Howe M. W. Graybiel A. M. (2012). Effects of dopamine depletion on LFP oscillations in striatum are task- and learning-dependent and selectively reversed by L-DOPA. Proc. Natl. Acad. Sci. U S A 109, 18126–18131 10.1073/pnas.1216403109 23074253 PMC3497773

[B142] Lenz F. A. Kwan H. C. Martin R. L. Tasker R. R. Dostrovsky J. O. Lenz Y. E. (1994). Single unit analysis of the human ventral thalamic nuclear group. Tremor-related activity in functionally identified cells. Brain 117 (Pt 3), 531–543 10.1093/brain/117.3.531 8032863

[B143] Lenz F. A. Normand S. L. Kwan H. C. Andrews D. Rowland L. H. Jones M. W. (1995). Statistical prediction of the optimal site for thalamotomy in parkinsonian tremor. Mov. Disord. 10, 318–328 10.1002/mds.870100315 7651450

[B145] Lenz F. A. Tasker R. R. Kwan H. C. Schnider S. Kwong R. Murayama Y. (1988). Single unit analysis of the human ventral thalamic nuclear group: correlation of thalamic “tremor cells” with the 3-6 Hz component of parkinsonian tremor. J. Neurosci. 8, 754–764 3346719 10.1523/JNEUROSCI.08-03-00754.1988 PMC6569249

[B144] Lenz F. A. Tasker R. R. Kwan H. C. Schnider S. Kwong R. Murphy J. T. (1985). Cross-correlation analysis of thalamic neurons and EMG activity in parkinsonian tremor. Appl. Neurophysiol. 48, 305–308 10.1159/000101148 3017210

[B146] Levy R. Ashby P. Hutchison W. D. Lang A. E. Lozano A. M. Dostrovsky J. O. (2002). Dependence of subthalamic nucleus oscillations on movement and dopamine in Parkinson’s disease. Brain 125, 1196–1209 10.1093/brain/awf128 12023310

[B147] Levy R. Hutchison W. D. Lozano A. M. Dostrovsky J. O. (2000). High-frequency synchronization of neuronal activity in the subthalamic nucleus of parkinsonian patients with limb tremor. J. Neurosci. 20, 7766–7775 11027240 10.1523/JNEUROSCI.20-20-07766.2000 PMC6772896

[B148] Lindvall O. Bjorklund A. Nobin A. Stenevi U. (1974). The adrenergic innervation of the rat thalamus as revealed by the glyoxylic acid fluorescence method. J. Comp. Neurol. 154, 317–347 10.1002/cne.901540307 4826099

[B149] Lisman J. E. (1997). Bursts as a unit of neural information: making unreliable synapses reliable. Trends Neurosci. 20, 38–43 10.1016/s0166-2236(96)10070-9 9004418

[B150] Llinas R. R. Steriade M. (2006). Bursting of thalamic neurons and states of vigilance. J. Neurophysiol. 95, 3297–3308 10.1152/jn.00166.2006 16554502

[B151] Luo M. Perkel D. J. (1999a). A GABAergic, strongly inhibitory projection to a thalamic nucleus in the zebra finch song system. J. Neurosci. 19, 6700–6711 10414999 10.1523/JNEUROSCI.19-15-06700.1999 PMC6782801

[B152] Luo M. Perkel D. J. (1999b). Long-range GABAergic projection in a circuit essential for vocal learning. J. Comp. Neurol. 403, 68–84 10.1002/(sici)1096-9861(19990105)403:1<68::aid-cne6>3.3.co;2-x 10075444

[B153] Luscher C. Huber K. M. (2010). Group 1 mGluR-dependent synaptic long-term depression: mechanisms and implications for circuitry and disease. Neuron 65, 445–459 10.1016/j.neuron.2010.01.016 20188650 PMC2841961

[B154] Macia F. Escola L. Guehl D. Michelet T. Bioulac B. Burbaud P. (2002). Neuronal activity in the monkey motor thalamus during bicuculline-induced dystonia. Eur. J. Neurosci. 15, 1353–1362 10.1046/j.1460-9568.2002.01964.x 11994129

[B155] MacPherson J. M. Rasmusson D. D. Murphy J. T. (1980). Activities of neurons in “motor” thalamus during control of limb movement in the primate. J. Neurophysiol. 44, 11–28 7420130 10.1152/jn.1980.44.1.11

[B156] Magill P. J. Bolam J. P. Bevan M. D. (2001). Dopamine regulates the impact of the cerebral cortex on the subthalamic nucleus-globus pallidus network. Neuroscience 106, 313–330 10.1016/s0306-4522(01)00281-0 11566503

[B157] Magnin M. Morel A. Jeanmonod D. (2000). Single-unit analysis of the pallidum, thalamus and subthalamic nucleus in parkinsonian patients. Neuroscience 96, 549–564 10.1016/s0306-4522(99)00583-7 10717435

[B158] Mainen Z. F. Sejnowski T. J. (1995). Reliability of spike timing in neocortical neurons. Science 268, 1503–1506 10.1126/science.7770778 7770778

[B159] Marceglia S. Foffani G. Bianchi A. M. Baselli G. Tamma F. Egidi M. (2006). Dopamine-dependent non-linear correlation between subthalamic rhythms in Parkinson’s disease. J. Physiol. 571, 579–591 10.1113/jphysiol.2005.100271 16410285 PMC1805793

[B160] Markham C. H. Brown W. J. Rand R. W. (1966). Stereotaxic lesions in Parkinson’s disease. Clinicopathological correlations. Arch. Neurol. 15, 480–497 10.1001/archneur.1966.00470170034004 5333735

[B161] Marsden J. F. Ashby P. Limousin-Dowsey P. Rothwell J. C. Brown P. (2000). Coherence between cerebellar thalamus, cortex and muscle in man: cerebellar thalamus interactions. Brain 123 ( Pt 7), 1459–1470 10.1093/brain/123.7.1459 10869057

[B162] Matsumura M. Sawaguchi T. Kubota K. (1992). GABAergic inhibition of neuronal activity in the primate motor and premotor cortex during voluntary movement. J. Neurophysiol. 68, 692–702 1432042 10.1152/jn.1992.68.3.692

[B163] Maurice N. Deniau J. M. Glowinski J. Thierry A. M. (1998). Relationships between the prefrontal cortex and the basal ganglia in the rat: physiology of the corticosubthalamic circuits. J. Neurosci. 18, 9539–9546 9801390 10.1523/JNEUROSCI.18-22-09539.1998 PMC6792878

[B164] Maurice N. Deniau J. M. Glowinski J. Thierry A. M. (1999). Relationships between the prefrontal cortex and the basal ganglia in the rat: physiology of the cortico-nigral circuits. J. Neurosci. 19, 4674–4681 10341265 10.1523/JNEUROSCI.19-11-04674.1999 PMC6782607

[B165] McCormick D. A. Huguenard J. R. (1992). A model of the electrophysiological properties of thalamocortical relay neurons. J. Neurophysiol. 68, 1384–1400 1331356 10.1152/jn.1992.68.4.1384

[B166] McFarland N. R. Haber S. N. (2002). Thalamic relay nuclei of the basal ganglia form both reciprocal and nonreciprocal cortical connections, linking multiple frontal cortical areas. J. Neurosci. 22, 8117–8132 12223566 10.1523/JNEUROSCI.22-18-08117.2002 PMC6758100

[B167] Medina L. Veenman C. L. Reiner A. (1997). Evidence for a possible avian dorsal thalamic region comparable to the mammalian ventral anterior, ventral lateral, and oral ventroposterolateral nuclei. J. Comp. Neurol. 384, 86–108 10.1002/(sici)1096-9861(19970721)384:1<86::aid-cne6>3.3.co;2-H 9214542

[B168] Middleton F. A. Strick P. L. (2000). Basal ganglia and cerebellar loops: motor and cognitive circuits. Brain Res. Brain Res. Rev. 31, 236–250 10.1016/s0165-0173(99)00040-5 10719151

[B170] Mink J. W. Thach W. T. (1991). Basal ganglia motor control. II. Late pallidal timing relative to movement onset and inconsistent pallidal coding of movement parameters. J. Neurophysiol. 65, 301–329 2016643 10.1152/jn.1991.65.2.301

[B171] Mure H. Hirano S. Tang C. C. Isaias I. U. Antonini A. Ma Y. (2011). Parkinson’s disease tremor-related metabolic network: characterization, progression, and treatment effects. Neuroimage 54, 1244–1253 10.1016/j.neuroimage.2010.09.028 20851193 PMC2997135

[B172] Mushiake H. Strick P. L. (1993). Preferential activity of dentate neurons during limb movements guided by vision. J. Neurophysiol. 70, 2660–2664 8120605 10.1152/jn.1993.70.6.2660

[B173] Mushiake H. Strick P. L. (1995). Pallidal neuron activity during sequential arm movements. J. Neurophysiol. 74, 2754–2758 8747231 10.1152/jn.1995.74.6.2754

[B174] Nagaseki Y. Shibazaki T. Hirai T. Kawashima Y. Hirato M. Wada H. (1986). Long-term follow-up results of selective VIM-thalamotomy. J. Neurosurg. 65, 296–302 10.3171/jns.1986.65.3.0296 3734879

[B175] Nakamura K. C. Sharott A. Magill P. J. (2012). Temporal coupling with cortex distinguishes spontaneous neuronal activities in identified basal ganglia-recipient and cerebellar-recipient zones of the motor thalamus. Cereb. Cortex [Epub ahead of print]. 10.1093/cercor/bhs287 23042738 PMC3862266

[B176] Nambu A. (2004). A new dynamic model of the cortico-basal ganglia loop. Prog. Brain Res. 143, 461–466 10.1016/s0079-6123(03)43043-4 14653188

[B177] Nambu A. (2007). Globus pallidus internal segment. Prog. Brain Res. 160, 135–150 10.1016/s0079-6123(06)60008-3 17499112

[B179] Nambu A. Yoshida S. Jinnai K. (1988). Projection on the motor cortex of thalamic neurons with pallidal input in the monkey. Exp. Brain Res. 71, 658–662 10.1007/bf00248759 3416976

[B180] Nambu A. Yoshida S. Jinnai K. (1990). Discharge patterns of pallidal neurons with input from various cortical areas during movement in the monkey. Brain Res. 519, 183–191 10.1016/0006-8993(90)90076-n 2397404

[B181] Nambu A. Yoshida S. Jinnai K. (1991). Movement-related activity of thalamic neurons with input from the globus pallidus and projection to the motor cortex in the monkey. Exp. Brain Res. 84, 279–284 10.1007/bf00231447 2065734

[B182] Nevet A. Morris G. Saban G. Arkadir D. Bergman H. (2007). Lack of spike-count and spike-time correlations in the substantia nigra reticulata despite overlap of neural responses. J. Neurophysiol. 98, 2232–2243 10.1152/jn.00190.2007 17699698

[B183] Nini A. Feingold A. Slovin H. Bergman H. (1995). Neurons in the globus pallidus do not show correlated activity in the normal monkey, but phase-locked oscillations appear in the MPTP model of parkinsonism. J. Neurophysiol. 74, 1800–1805 8989416 10.1152/jn.1995.74.4.1800

[B184] Obeso J. A. Rodriguez-Oroz M. C. Rodriguez M. Lanciego J. L. Artieda J. Gonzalo N. (2000). Pathophysiology of the basal ganglia in Parkinson’s disease. Trends Neurosci. 23, S8–S19 10.1016/S1471-1931(00)00028-8 11052215

[B185] Ohyama T. Nores W. L. Murphy M. Mauk M. D. (2003). What the cerebellum computes. Trends Neurosci. 26, 222–227 10.1016/s0166-2236(03)00054-7 12689774

[B186] Okun M. S. Vitek J. L. (2004). Lesion therapy for Parkinson’s disease and other movement disorders: update and controversies. Mov. Disord. 19, 375–389 10.1002/mds.20037 15077235

[B187] Pahwa R. Lyons K. E. Wilkinson S. B. Simpson R. K. Jr. Ondo W. G. (2006). Long-term evaluation of deep brain stimulation of the thalamus. J. Neurosurg. 104, 506–512 10.3171/jns.2006.104.4.506 16619653

[B188] Paradiso G. Cunic D. Saint-Cyr J. A. Hoque T. Lozano A. M. Lang A. E. (2004). Involvement of human thalamus in the preparation of self-paced movement. Brain 127, 2717–2731 10.1093/brain/awh288 15329354

[B189] Pare D. Hazrati L. N. Parent A. Steriade M. (1990). Substantia nigra pars reticulata projects to the reticular thalamic nucleus of the cat: a morphological and electrophysiological study. Brain Res. 535, 139–146 10.1016/0006-8993(90)91832-2 1705469

[B190] Pare D. Steriade M. Deschenes M. Oakson G. (1987). Physiological characteristics of anterior thalamic nuclei, a group devoid of inputs from reticular thalamic nucleus. J. Neurophysiol. 57, 1669–1685 3037038 10.1152/jn.1987.57.6.1669

[B191] Parr-Brownlie L. C. Hyland B. I. (2005). Bradykinesia induced by dopamine D2 receptor blockade is associated with reduced motor cortex activity in the rat. J. Neurosci. 25, 5700–5709 10.1523/jneurosci.0523-05.2005 15958736 PMC6724886

[B192] Parr-Brownlie L. C. Poloskey S. L. Flanagan K. K. Eisenhofer G. Bergstrom D. A. Walters J. R. (2007). Dopamine lesion-induced changes in subthalamic nucleus activity are not associated with alterations in firing rate or pattern in layer V neurons of the anterior cingulate cortex in anesthetized rats. Eur. J. Neurosci. 26, 1925–1939 10.1111/j.1460-9568.2007.05814.x 17897398

[B193] Pasquereau B. Turner R. S. (2011). Primary motor cortex of the parkinsonian monkey: differential effects on the spontaneous activity of pyramidal tract-type neurons. Cereb. Cortex 21, 1362–1378 10.1093/cercor/bhq217 21045003 PMC3097989

[B194] Paz J. T. Chavez M. Saillet S. Deniau J. M. Charpier S. (2007). Activity of ventral medial thalamic neurons during absence seizures and modulation of cortical paroxysms by the nigrothalamic pathway. J. Neurosci. 27, 929–941 10.1523/jneurosci.4677-06.2007 17251435 PMC6672924

[B195] Pazo J. H. Barcelo A. C. Bellantonio E. Pazo V. C. Almarares N. (2013). Electrophysiologic study of globus pallidus projections to the thalamic reticular nucleus. Brain Res. Bull. 94, 82–89 10.1016/j.brainresbull.2013.02.009 23500178

[B196] Percheron G. Francois C. Talbi B. Yelnik J. Fenelon G. (1996). The primate motor thalamus. Brain Res. Brain Res. Rev. 22, 93–181 10.1016/s0165-0173(96)00003-3 8883918

[B197] Perret E. (1968). Simple motor performance of patients with Parkinson’s disease before and after a surgical lesion in the thalamus. J. Neurol. Neurosurg. Psychiatry 31, 284–290 10.1136/jnnp.31.3.284 4879171 PMC496357

[B198] Perret E. Eggenberger E. Siegfried J. (1970). Simple and complex finger movement performance of patients with Parkinsonism before and after a unilateral stereotaxic thalamotomy. J. Neurol. Neurosurg. Psychiatry 33, 16–21 10.1136/jnnp.33.1.16 4907277 PMC493402

[B199] Person A. L. Perkel D. J. (2005). Unitary IPSPs drive precise thalamic spiking in a circuit required for learning. Neuron 46, 129–140 10.1016/j.neuron.2004.12.057 15820699

[B200] Person A. L. Perkel D. J. (2007). Pallidal neuron activity increases during sensory relay through thalamus in a songbird circuit essential for learning. J. Neurosci. 27, 8687–8698 10.1523/jneurosci.2045-07.2007 17687046 PMC6672941

[B201] Pessiglione M. Guehl D. Rolland A. S. Francois C. Hirsch E. C. Feger J. (2005). Thalamic neuronal activity in dopamine-depleted primates: evidence for a loss of functional segregation within basal ganglia circuits. J. Neurosci. 25, 1523–1531 10.1523/jneurosci.4056-04.2005 15703406 PMC6725984

[B202] Postupna N. O. Anderson M. (2002). Bursting discharge in monkey thalamus. 32nd Annual meeting of the Society for Neuroscience, 62.11.

[B203] Quallo M. M. Kraskov A. Lemon R. N. (2012). The activity of primary motor cortex corticospinal neurons during tool use by macaque monkeys. J. Neurosci. 32, 17351–17364 10.1523/JNEUROSCI.1009-12.2012 23197726 PMC3678117

[B204] Raeva S. N. Vainberg N. A. Tikhonov Yu Tsetlin I. M. (1998). Analysis of evoked activity patterns of human thalamic ventrolateral neurons during verbally ordered voluntary movements. Neuroscience 88, 377–392 10.1016/s0306-4522(98)00230-9 10197761

[B205] Raman I. M. Bean B. P. (1997). Resurgent sodium current and action potential formation in dissociated cerebellar Purkinje neurons. J. Neurosci. 17, 4517–4526 9169512 10.1523/JNEUROSCI.17-12-04517.1997 PMC6573347

[B206] Raz A. Frechter-Mazar V. Feingold A. Abeles M. Vaadia E. Bergman H. (2001). Activity of pallidal and striatal tonically active neurons is correlated in mptp-treated monkeys but not in normal monkeys. J. Neurosci. 21, 1–5 11157099 10.1523/JNEUROSCI.21-03-j0006.2001 PMC6762319

[B207] Raz A. Vaadia E. Bergman H. (2000). Firing patterns and correlations of spontaneous discharge of pallidal neurons in the normal and the tremulous 1-methyl-4-phenyl-1,2,3,6-tetrahydropyridine vervet model of parkinsonism. J. Neurosci. 20, 8559–8571 11069964 10.1523/JNEUROSCI.20-22-08559.2000 PMC6773163

[B208] Redgrave P. Rodriguez M. Smith Y. Rodriguez-Oroz M. C. Lehericy S. Bergman H. (2010). Goal-directed and habitual control in the basal ganglia: implications for Parkinson’s disease. Nat. Rev. Neurosci. 11, 760–772 10.1038/nrn2915 20944662 PMC3124757

[B209] Rinvik E. Grofova I. (1974). Cerebellar projections to the nuclei ventralis lateralis and ventralis anterior thalami. Experimental electron microscopical and light microscopical studies in the cat. Anat. Embryol. (Berl) 146, 95–111 10.1007/BF00341384 4618725

[B210] Rivner M. Sutin J. (1981). Locus coeruleus modulation of the motor thalamus: inhibition in nuclei ventralis lateralis and ventralis anterior. Exp. Neurol. 73, 651–673 10.1016/0014-4886(81)90203-x 7262261

[B212] Rouiller E. M. Tanne J. Moret V. Boussaoud D. (1999). Origin of thalamic inputs to the primary, premotor, and supplementary motor cortical areas and to area 46 in macaque monkeys: a multiple retrograde tracing study. J. Comp. Neurol. 409, 131–152 10.1002/(sici)1096-9861(19990621)409:1<131::aid-cne10>3.0.co;2-a 10363716

[B211] Rouiller E. Wannier T. Morel A. (2003). The dual patten of corticothalamic projection of premotor cortex in macaque monkeys. Thalamus Relat. Syst. 2, 189–197 10.1016/s1472-9288(03)00019-0

[B213] Rouiller E. M. Tanne J. Moret V. Kermadi I. Boussaoud D. Welker E. (1998). Dual morphology and topography of the corticothalamic terminals originating from the primary, supplementary motor, and dorsal premotor cortical areas in macaque monkeys. J. Comp. Neurol. 396, 169–185 10.1002/(sici)1096-9861(19980629)396:2<169::aid-cne3>3.0.co;2-z 9634140

[B214] Rovo Z. Ulbert I. Acsady L. (2012). Drivers of the primate thalamus. J. Neurosci. 32, 17894–17908 10.1523/jneurosci.2815-12.2012 23223308 PMC3672843

[B215] Rowland N. C. Goldberg J. A. Jaeger D. (2010). Cortico-cerebellar coherence and causal connectivity during slow-wave activity. Neuroscience 166, 698–711 10.1016/j.neuroscience.2009.12.048 20036719 PMC2823967

[B216] Rubin J. E. Mcintyre C. C. Turner R. S. Wichmann T. (2012). Basal ganglia activity patterns in parkinsonism and computational modeling of their downstream effects. Eur. J. Neurosci. 36, 2213–2228 10.1111/j.1460-9568.2012.08108.x 22805066 PMC3400124

[B217] Sakai S. T. Grofova I. Bruce K. (1998). Nigrothalamic projections and nigrothalamocortical pathway to the medial agranular cortex in the rat: single- and double-labeling light and electron microscopic studies. J. Comp. Neurol. 391, 506–525 10.1002/(sici)1096-9861(19980222)391:4<506::aid-cne7>3.0.co;2-4 9486828

[B218] Sakai S. T. Inase M. Tanji J. (1996). Comparison of cerebellothalamic and pallidothalamic projections in the monkey (Macaca fuscata): a double anterograde labeling study. J. Comp. Neurol. 368, 215–228 10.1002/(sici)1096-9861(19960429)368:2<215::aid-cne4>3.0.co;2-6 8725303

[B219] Sakai S. T. Stepniewska I. Qi H. X. Kaas J. H. (2000). Pallidal and cerebellar afferents to pre-supplementary motor area thalamocortical neurons in the owl monkey: a multiple labeling study. J. Comp. Neurol. 417, 164–180 10.1002/(sici)1096-9861(20000207)417:2<164::aid-cne3>3.3.co;2-y 10660895

[B220] Sanes J. N. Donoghue J. P. (1993). Oscillations in local field potentials of the primate motor cortex during voluntary movement. Proc. Natl. Acad. Sci. U S A 90, 4470–4474 10.1073/pnas.90.10.4470 8506287 PMC46533

[B221] Sawaguchi T. Matsumura M. Kubota K. (1989). Depth distribution of neuronal activity related to a visual reaction time task in the monkey prefrontal cortex. J. Neurophysiol. 61, 435–446 2918365 10.1152/jn.1989.61.2.435

[B222] Sawyer S. F. Tepper J. M. Groves P. M. (1994a). Cerebellar-responsive neurons in the thalamic ventroanterior-ventrolateral complex of rats: light and electron microscopy. Neuroscience 63, 725–745 10.1016/0306-4522(94)90518-5 7898673

[B223] Sawyer S. F. Young S. J. Groves P. M. Tepper J. M. (1994b). Cerebellar-responsive neurons in the thalamic ventroanterior-ventrolateral complex of rats: in vivo electrophysiology. Neuroscience 63, 711–724 10.1016/0306-4522(94)90517-7 7898672

[B224] Schmied A. Benita M. Conde H. Dormont J. F. (1979). Activity of ventrolateral thalamic neurons in relation to a simple reaction time task in the cat. Exp. Brain Res. 36, 285–300 10.1007/bf00238912 488203

[B225] Schneider J. S. Rothblat D. S. (1996). Alterations in intralaminar and motor thalamic physiology following nigrostriatal dopamine depletion. Brain Res. 742, 25–33 10.1016/s0006-8993(96)00988-2 9117401

[B226] Schomburg E. W. Anastassiou C. A. Buzsaki G. Koch C. (2012). The spiking component of oscillatory extracellular potentials in the rat hippocampus. J. Neurosci. 32, 11798–11811 10.1523/jneurosci.0656-12.2012 22915121 PMC3459239

[B227] Sharott A. Magill P. J. Harnack D. Kupsch A. Meissner W. Brown P. (2005). Dopamine depletion increases the power and coherence of beta-oscillations in the cerebral cortex and subthalamic nucleus of the awake rat. Eur. J. Neurosci. 21, 1413–1422 10.1111/j.1460-9568.2005.03973.x 15813951

[B228] Sherman S. M. (2001). Tonic and burst firing: dual modes of thalamocortical relay. Trends Neurosci. 24, 122–126 10.1016/s0166-2236(00)01714-8 11164943

[B229] Sherman S. M. (2007). The thalamus is more than just a relay. Curr. Opin. Neurobiol. 17, 417–422 10.1016/j.conb.2007.07.003 17707635 PMC2753250

[B230] Sherman S. M. Guillery R. W. (1998). On the actions that one nerve cell can have on another: distinguishing “drivers” from “modulators”. Proc. Natl. Acad. Sci. U S A 95, 7121–7126 10.1073/pnas.95.12.7121 9618549 PMC22761

[B231] Sherman S. M. Guillery R. W. (2006). Exploring the Thalamus and its Role in Cortical Function. 2nd Edn Cambridge, MA: MIT Press

[B232] Sherman S. M. Guillery R. W. (2011). Distinct functions for direct and transthalamic corticocortical connections. J. Neurophysiol. 106, 1068–1077 10.1152/jn.00429.2011 21676936

[B233] Shinoda Y. Futami T. Kano M. (1985). Synaptic organization of the cerebello-thalamo-cerebral pathway in the cat. II. Input-output organization of single thalamocortical neurons in the ventrolateral thalamus. Neurosci. Res. 2, 157–180 10.1016/0168-0102(85)90010-0 2991825

[B234] Sieb R. A. (1989). Proposed mechanisms for cerebellar coordination, stabilization and monitoring of movements and posture. Med. Hypotheses 28, 225–232 10.1016/0306-9877(89)90076-5 2739591

[B235] Sirota M. G. Swadlow H. A. Beloozerova I. N. (2005). Three channels of corticothalamic communication during locomotion. J. Neurosci. 25, 5915–5925 10.1523/jneurosci.0489-05.2005 15976080 PMC6724793

[B236] Smith G. D. Sherman S. M. (2002). Detectability of excitatory versus inhibitory drive in an integrate-and-fire-or-burst thalamocortical relay neuron model. J. Neurosci. 22, 10242–10250 12451125 10.1523/JNEUROSCI.22-23-10242.2002 PMC6758741

[B237] Sommer M. A. (2003). The role of the thalamus in motor control. Curr. Opin. Neurobiol. 13, 663–670 10.1016/j.conb.2003.10.014 14662366

[B238] Starr M. S. Summerhayes M. (1983a). Role of the ventromedial nucleus of the thalamus in motor behaviour–I. Effects of focal injections of drugs. Neuroscience 10, 1157–1169 10.1016/0306-4522(83)90106-9 6320046

[B239] Starr M. S. Summerhayes M. (1983b). Role of the ventromedial nucleus of the thalamus in motor behaviour–II. Effects of lesions. Neuroscience 10, 1171–1183 10.1016/0306-4522(83)90107-0 6320047

[B240] Steriade M. (2006). Grouping of brain rhythms in corticothalamic systems. Neuroscience 137, 1087–1106 10.1016/j.neuroscience.2005.10.029 16343791

[B241] Steriade M. Apostol V. Oakson G. (1971). Control of unitary activities in cerebellothalamic pathway during wakefulness and synchronized sleep. J. Neurophysiol. 34, 389–413 4327048 10.1152/jn.1971.34.3.389

[B242] Steriade M. Pare D. Parent A. Smith Y. (1988). Projections of cholinergic and non-cholinergic neurons of the brainstem core to relay and associational thalamic nuclei in the cat and macaque monkey. Neuroscience 25, 47–67 10.1016/0306-4522(88)90006-1 3393286

[B243] Strick P. L. (1976). Activity of ventrolateral thalamic neurons during arm movement. J. Neurophysiol. 39, 1032–1044 824408 10.1152/jn.1976.39.5.1032

[B244] Tai C. H. Boraud T. Bezard E. Bioulac B. Gross C. Benazzouz A. (2003). Electrophysiological and metabolic evidence that high-frequency stimulation of the subthalamic nucleus bridles neuronal activity in the subthalamic nucleus and the substantia nigra reticulata. FASEB J. 17, 1820–1830 10.1096/fj.03-0163com 14519661

[B245] Tanibuchi I. Kitano H. Jinnai K. (2009). Substantia nigra output to prefrontal cortex via thalamus in monkeys. I. Electrophysiological identification of thalamic relay neurons. J. Neurophysiol. 102, 2933–2945 10.1152/jn.91287.2008 19692504

[B246] Tarr T. B. Dittrich M. Meriney S. D. (2013). Are unreliable release mechanisms conserved from NMJ to CNS? Trends Neurosci. 36, 14–22 10.1016/j.tins.2012.09.009 23102681 PMC4076818

[B247] Thach W. T. (1978). Correlation of neural discharge with pattern and force of muscular activity, joint position, and direction of intended next movement in motor cortex and cerebellum. J. Neurophysiol. 41, 654–676 96223 10.1152/jn.1978.41.3.654

[B248] Thach W. T. Goodkin H. P. Keating J. G. (1992). The cerebellum and the adaptive coordination of movement. Annu. Rev. Neurosci. 15, 403–442 10.1146/annurev.neuro.15.1.403 1575449

[B249] Trouche E. Beaubaton D. (1980). Initiation of a goal-directed movement in the monkey. Role of the cerebellar dentate nucleus. Exp. Brain Res. 40, 311–321 10.1007/bf00237796 7428885

[B250] Tseng K. Y. Kasanetz F. Kargieman L. Riquelme L. A. Murer M. G. (2001). Cortical slow oscillatory activity is reflected in the membrane potential and spike trains of striatal neurons in rats with chronic nigrostriatal lesions. J. Neurosci. 21, 6430–6439 11487667 10.1523/JNEUROSCI.21-16-06430.2001 PMC6763136

[B251] Turner R. S. Anderson M. E. (1997). Pallidal discharge related to the kinematics of reaching movements in two dimensions. J. Neurophysiol. 77, 1051–1074 9084582 10.1152/jn.1997.77.3.1051

[B252] Turner R. S. Anderson M. E. (2005). Context-dependent modulation of movement-related discharge in the primate globus pallidus. J. Neurosci. 25, 2965–2976 10.1523/jneurosci.4036-04.2005 15772356 PMC6725146

[B253] Turner R. S. Desmurget M. (2010). Basal ganglia contributions to motor control: a vigorous tutor. Curr. Opin. Neurobiol. 20, 704–716 10.1016/j.conb.2010.08.022 20850966 PMC3025075

[B254] Ueki A. (1983). The mode of nigro-thalamic transmission investigated with intracellular recording in the cat. Exp. Brain Res. 49, 116–124 10.1007/bf00235546 6305696

[B255] Ueki A. Uno M. Anderson M. Yoshida M. (1977). Monosynaptic inhibition of thalamic neurons produced by stimulation of the substantia nigra. Experientia 33, 1480–1482 10.1007/bf01918820 923717

[B256] Uno M. Ozawa N. Yoshida M. (1978). The mole of pallido-thalamic transmission investigated with intracellular recording from cat thalamus. Exp. Brain Res. 33, 493–507 10.1007/bf00235570 215434

[B257] Uno M. Yoshida M. Hirota I. (1970). The mode of cerebello-thalamic relay transmission investigated with intracellular recodrings from cells of the ventrolateral nucleus of ’cat’s thalamus. Exp. Brain Res. 10, 121–139 10.1007/bf00234726 4314010

[B258] Ushimaru M. Ueta Y. Kawaguchi Y. (2012). Differentiated participation of thalamocortical subnetworks in slow/spindle waves and desynchronization. J. Neurosci. 32, 1730–1746 10.1523/JNEUROSCI.4883-11.2012 22302813 PMC6703373

[B259] Vaadia E. Haalman I. Abeles M. Bergman H. Prut Y. Slovin H. (1995). Dynamics of neuronal interactions in monkey cortex in relation to behavioural events. Nature 373, 515–518 10.1038/373515a0 7845462

[B260] van Someren E. J. van Gool W. A. Vonk B. F. Mirmiran M. Speelman J. D. Bosch D. A. (1993). Ambulatory monitoring of tremor and other movements before and after thalamotomy: a new quantitative technique. J. Neurol. Sci. 117, 16–23 10.1016/0022-510x(93)90148-r 8410051

[B261] Viitanen T. Ruusuvuori E. Kaila K. Voipio J. (2010). The K+-Cl cotransporter KCC2 promotes GABAergic excitation in the mature rat hippocampus. J. Physiol. 588, 1527–1540 10.1113/jphysiol.2009.181826 20211979 PMC2876807

[B262] Vitek J. L. Ashe J. DeLong M. R. Alexander G. E. (1994). Physiologic properties and somatotopic organization of the primate motor thalamus. J. Neurophysiol. 71, 1498–1513 8035231 10.1152/jn.1994.71.4.1498

[B263] Volkmann J. Joliot M. Mogilner A. Ioannides A. A. Lado F. Fazzini E. (1996). Central motor loop oscillations in parkinsonian resting tremor revealed by magnetoencephalography. Neurology 46, 1359–1370 10.1212/wnl.46.5.1359 8628483

[B264] Voloshin M. Lukhanina E. P. Kolomietz B. P. Prokopenko V. F. Rodionov V. A. (1994). Electrophysiological investigation of thalamic neuronal mechanisms of motor disorders in parkinsonism: an influence of D2ergic transmission blockade on excitation and inhibition of relay neurons in motor thalamic nuclei of cat. Neuroscience 62, 771–781 10.1016/0306-4522(94)90475-8 7870305

[B265] Walters J. R. Hu D. Itoga C. A. Parr-Brownlie L. C. Bergstrom D. A. (2007). Phase relationships support a role for coordinated activity in the indirect pathway in organizing slow oscillations in basal ganglia output after loss of dopamine. Neuroscience 144, 762–776 10.1016/j.neuroscience.2006.10.006 17112675 PMC3354994

[B266] Weinberger M. Mahant N. Hutchison W. D. Lozano A. M. Moro E. Hodaie M. (2006). Beta oscillatory activity in the subthalamic nucleus and its relation to dopaminergic response in Parkinson’s disease. J. Neurophysiol. 96, 3248–3256 10.1152/jn.00697.2006 17005611

[B267] Wichmann T. Bergman H. Starr P. A. Subramanian T. Watts R. L. DeLong M. R. (1999). Comparison of MPTP-induced changes in spontaneous neuronal discharge in the internal pallidal segment and in the substantia nigra pars reticulata in primates. Exp. Brain Res. 125, 397–409 10.1007/s002210050696 10323285

[B268] Wichmann T. Kliem M. A. (2004). Neuronal activity in the primate substantia nigra pars reticulata during the performance of simple and memory-guided elbow movements. J. Neurophysiol. 91, 815–827 10.1152/jn.01180.2002 14762150

[B269] Wickens J. Hyland B. Anson G. (1994). Cortical cell assemblies: a possible mechanism for motor programs. J. Mot. Behav. 26, 66–82 10.1080/00222895.1994.9941663 15753061

[B270] Williams M. N. Faull R. L. (1987). The distribution and morphology of identified thalamocortical projection neurons and glial cells with reference to the question of interneurons in the ventrolateral nucleus of the rat thalamus. Neuroscience 21, 767–780 10.1016/0306-4522(87)90036-4 3627434

[B271] Williams D. Tijssen M. Van Bruggen G. Bosch A. Insola A. Di Lazzaro V. (2002). Dopamine-dependent changes in the functional connectivity between basal ganglia and cerebral cortex in humans. Brain 125(Pt 7), 1558–1569 10.1093/brain/awf156 12077005

[B272] Wullner U. Klockgether T. Schwarz M. Sontag K. H. (1987). Behavioral actions of baclofen in the rat ventromedial thalamic nucleus: antagonism by delta-aminovalerate. Brain Res. 422, 129–136 10.1016/0006-8993(87)90547-6 3676775

[B273] Xia Z. Storm D. R. (2005). The role of calmodulin as a signal integrator for synaptic plasticity. Nat. Rev. Neurosci. 6, 267–276 10.1038/nrn1647 15803158

[B274] Xiao D. Zikopoulos B. Barbas H. (2009). Laminar and modular organization of prefrontal projections to multiple thalamic nuclei. Neuroscience 161, 1067–1081 10.1016/j.neuroscience.2009.04.034 19376204 PMC2700123

[B275] Xu W. Morishita W. Buckmaster P. S. Pang Z. P. Malenka R. C. Sudhof T. C. (2012). Distinct neuronal coding schemes in memory revealed by selective erasure of fast synchronous synaptic transmission. Neuron 73, 990–1001 10.1016/j.neuron.2011.12.036 22405208 PMC3319466

[B276] Yamamoto T. Katayama Y. Kano T. Kobayashi K. Oshima H. Fukaya C. (2004). Deep brain stimulation for the treatment of parkinsonian, essential, and poststroke tremor: a suitable stimulation method and changes in effective stimulation intensity. J. Neurosurg. 101, 201–209 10.3171/jns.2004.101.2.0201 15309909

[B277] Yamamoto T. Kishimoto Y. Yoshikawa H. Oka H. (1991). Intracellular recordings from rat thalamic VL neurons: a study combined with intracellular staining. Exp. Brain Res. 87, 245–253 10.1007/bf00231841 1769380

[B278] Yamamoto T. Noda T. Miyata M. Nishimura Y. (1984). Electrophysiological and morphological studies on thalamic neurons receiving entopedunculo- and cerebello-thalamic projections in the cat. Brain Res. 301, 231–242 10.1016/0006-8993(84)91091-6 6329450

[B279] Zirh T. A. Lenz F. A. Reich S. G. Dougherty P. M. (1998). Patterns of bursting occurring in thalamic cells during parkinsonian tremor. Neuroscience 83, 107–121 10.1016/s0306-4522(97)00295-9 9466402

[B280] Zucker R. S. Regehr W. G. (2002). Short-term synaptic plasticity. Annu. Rev. Physiol. 64, 355–405 10.1146/annurev.physiol.64.092501.114547 11826273

